# Decrypting Strong and Weak Single-Walled Carbon Nanotubes Interactions with Mitochondrial Voltage-Dependent Anion Channels Using Molecular Docking and Perturbation Theory

**DOI:** 10.1038/s41598-017-13691-8

**Published:** 2017-10-16

**Authors:** Michael González-Durruthy, Adriano V. Werhli, Vinicius Seus, Karina S. Machado, Alejandro Pazos, Cristian R. Munteanu, Humberto González-Díaz, José M. Monserrat

**Affiliations:** 1 Institute of Biological Sciences (ICB)- Federal University of Rio Grande - FURG, Postgraduate Program in Physiological Sciences, Cx. P. 474, CEP 96200-970 Rio Grande, RS Brazil; 2 Center of Computational Sciences (C3)- Federal University of Rio Grande - FURG, Cx. P. 474, CEP 96200-970 Rio Grande, RS Brazil; 3 Biomedical Research Institute of A Coruña (INIBIC), University Hospital Complex of A Coruña (CHUAC), A Coruña, 15006 Spain; 4 RNASA-IMEDIR, Computer Science Faculty, University of A Coruña, Campus de Elviña s/n, 15071 A Coruña, Spain; 5 Department of Organic Chemistry II, University of the Basque Country UPV/EHU, 48940 Leioa, Spain; 6 IKERBASQUE, Basque Foundation for Science, 48011 Bilbao, Spain

## Abstract

The current molecular docking study provided the Free Energy of Binding (FEB) for the interaction (nanotoxicity) between VDAC mitochondrial channels of three species (VDAC1-*Mus musculus*, VDAC1*-Homo sapiens*, VDAC2*-Danio rerio*) with SWCNT-H, SWCNT-OH, SWCNT-COOH carbon nanotubes. The general results showed that the FEB values were statistically more negative (p < 0.05) in the following order**: (**SWCNT-VDAC2-*Danio rerio*) > (SWCNT-VDAC1-*Mus musculus*) > (SWCNT-VDAC1-*Homo sapiens*) > (ATP-VDAC). More negative FEB values for SWCNT-COOH and OH were found in VDAC2-*Danio rerio* when compared with VDAC1-*Mus musculus* and VDAC1*-Homo sapiens* (p < 0.05). In addition, *a* significant correlation (0.66 > r^2^ > 0.97) was observed between *n*-Hamada index and VDAC nanotoxicity (or FEB) for the zigzag topologies of SWCNT-COOH and SWCNT-OH. Predictive Nanoparticles-Quantitative-Structure Binding-Relationship models (nano-QSBR) for strong and weak SWCNT-VDAC docking interactions were performed using Perturbation Theory, regression and classification models. Thus, 405 SWCNT-VDAC interactions were predicted using a nano-PT-QSBR classifications model with high accuracy, specificity, and sensitivity (73–98%) in training and validation series, and a maximum AUROC value of 0.978. In addition, the best regression model was obtained with Random Forest (R^2^ of 0.833, RMSE of 0.0844), suggesting an excellent potential to predict SWCNT-VDAC channel nanotoxicity. All study data are available at https://doi.org/10.6084/m9.figshare.4802320.v2.

## Introduction

VDAC is the most abundant and highly conserved channel-protein in outer mitochondrial membrane of cells from all eukaryotic kingdoms including human (*Homo sapiens*). VDAC is the main communication route between this organelle and cytosol, generating the mitochondrial membrane potential, sucrose exchange, mitochondrial [Ca^2+^] dynamics and ATP-efflux between these two cellular compartments^1–4^. Besides, VDAC is an essential component of the induced-structure of mitochondrial permeability transition pore (MPTP-VDAC), a multi-protein complex that is directly involved in mitochondrial dysfunction (apoptosis)^5–9^.

Carbon nanotubes (CNT) are nanomaterials considered for biomedical applications due to their flexible nature and versatility for chemical functionalization/oxidation (e.g. OH, COOH)^10,11^. More specifically, single-walled carbon nanotubes (SWCNT) have rapidly become one of the most widely studied nanomaterials, based on their unique physico-chemical properties that allow their potential use in new nanomedicine applications like pharmaceutical excipients for the design of several drug delivery systems^10,11^.

The VDAC channel inhibition by SWCNT could be an attractive therapeutic strategy to induce mitotoxicity based on specific VDAC-modulation. Following this idea, the chemo-informatics tools based on Docking Simulation (DS)^12^ appears to be an efficient strategy for the potential nanotoxicity prediction and SWCNT environmental impact. The use of Docking Simulation coupled to a Virtual Screening Framework (DS-VSF)^12,13^ is a powerful new platform for the rational design of a new SWCNT before its mass production, allowing for the computational interaction analysis of a large volume of SWCNT designs with key molecular targets^14,15^. Several *in vitro* studies have demonstrated that SWCNTs exert their cytotoxicity mechanism after their accumulation in the mitochondria matrix^16,17^.

In this context, chemoinformatics algorithms may be also very useful to predict carbon nanomaterial structure-activity relationships (QSAR models). Specifically, the term Quantitative Structure-Binding Relationships (QSBR) model is used for ligand-protein interactions. In the particular case of Nanoparticles we can talk about Nano-QSAR (NQSAR) or Nano-QSBR (NQSBR) models, by analogy. In general, these NQSAR/NQSBR models use as input physico-chemical properties of nanoparticles (nanodescriptors) to predict their biological activity, toxicity, and/or target-binding affinity^18–29^. The main assumption of classic QSAR algorithms in general form is that the similar structures (ligands) have similar properties. Consequently, small structural changes (“perturbations”) should correlate linearly with small changes in the values of their properties biological (endpoints). NQSAR/NQSBR models based on Perturbation Theory use as a first parameter the value of an exact solution to the problem. This first parameter represents a known value of biological property used as reference value (or an expected value of this endpoint). After that, the PT-NQSAR model add small corrections (functions of nanodescriptors for one specific case) to predict a solution to a related problem^18,30–32^. In principle, there is a large variety of data analysis techniques that have proven to be effective in QSAR/QSBR modeling in general (including NQSAR and PT-NQSAR models). Examples of these techniques are: Linear Discriminant Analysis (LDA), Neural Networks (NN), Random Forest (RF), *etc*.^33–38^. Last, we should note that, NQSAR/NQSBR algorithms should have the ability of feature selection (FS) to exclude redundant nanodescriptors before NQSAR model building^39,40^. Specifically, (*n*, *m*)*-*Hamada indices (related to SWCNT-chiral topology and SWCNT-diameter) may become interesting candidates to input variables in NQSAR/NQSBR studies^18^. The electro-topological and constitutional SWCNT nanodescriptors for pristine SWCNT and oxidized SWCNT (SWCNT-OH, SWCNT-COOH), based on the information of SWCNT-periodic properties^41–45^, have not yet been considered from the perspective of NQSAR with respect to their relevant interactions with mitochondrial channels, such as voltage-dependent anion channel (VDAC).

In this context, the main objective of this study was to evaluate *in silico* interactions (or mitochondrial channel nanotoxicity) between pristine and oxidized SWCNT and VDAC from different species (VDAC1-*Mus musculus*, VDAC1-*Homo sapiens*, and VDAC2-*Danio rerio*) using DS-VSF Chemoinformatic tools. These models are expected to be able to predict the strength of SWCNT-interaction and to quantify the structural requirements of SWCNT family governing the strong or weak SWCNT-VDAC interactions. In this sense, after docking studies we developed a new PT-NQSBR model using as input SWCNT-structural properties (Hamada indices, *etc*.) and *Free Energy of Binding* (*FEB* values), obtained after DS to perform a PT-NQSBR model based on LDA technique. LDA was implemented because it allows explaining the linear relationship between the input variables (SWCNT-structural properties) and ‘nanotoxicological endpoint’ (FEB values). To verify potential errors in the linear relationship hypothesis on SWCNT nanodescriptors/VDAC channel nanotoxicity, new non-linear classification and regression models were proposed. These non-linear PT-NQSBR models are based on machine learning algorithms implemented on Weka and RRegrs (R package)^33–38^. The present work could pave the way for the use of chemo-informatics tools based on SWCNT-ligand and mitotarget docking interactions for making regulatory decisions in Nanotoxicology, allowing the prediction of potential human health impact and environment risks.

## Results and Discussion

### Mechanistic interpretation of molecular docking results

**Figure 1:**
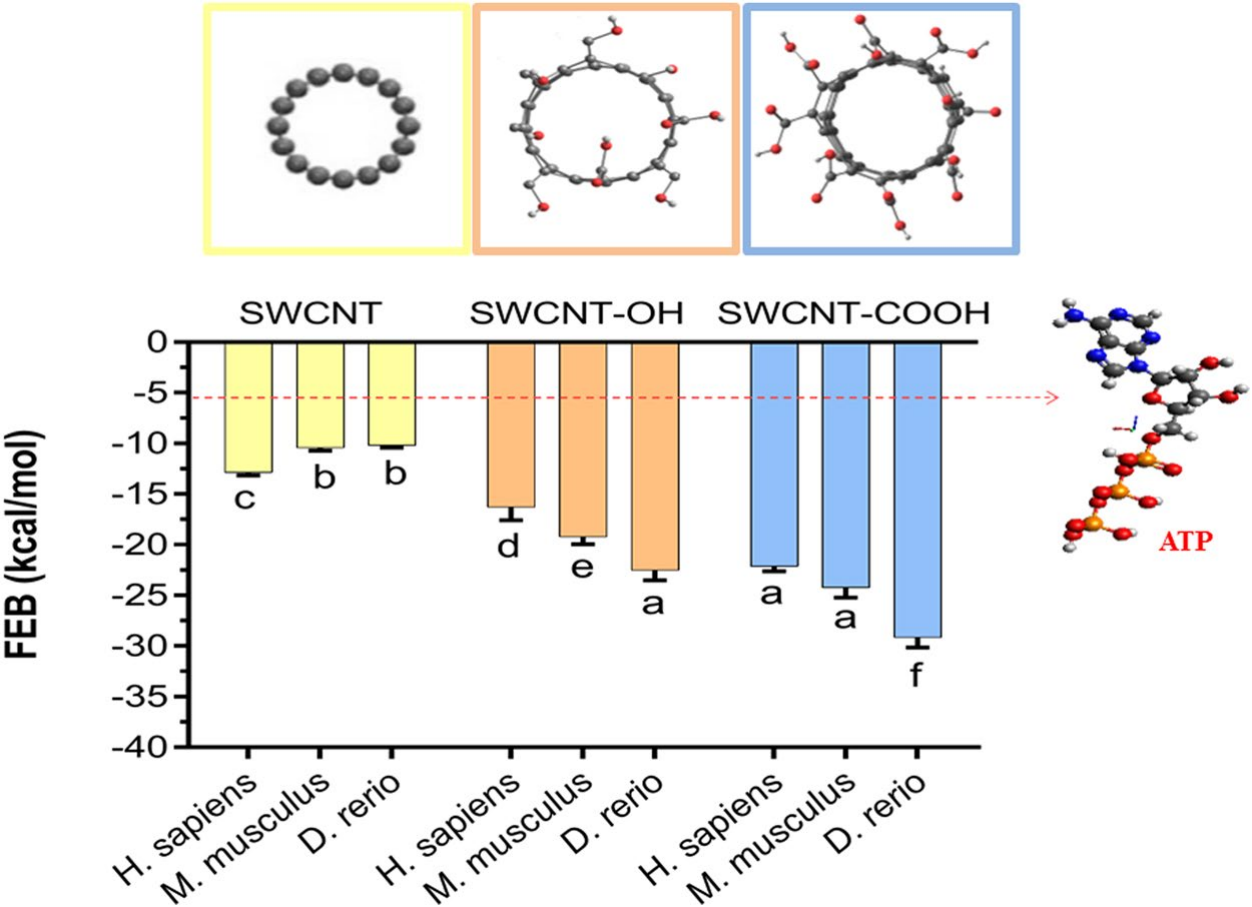
Free energy binding (FEB, in kcal mol^−1^) of mitochondrial voltage-dependent anion channel (VDAC) with pristine, hydroxylated and carboxylated single-walled carbon nanotubes (SWCNT, SWCNT-OH and SWCNT-COOH, respectively). Each FEB-value is expressed in terms of the mean ±1 error standard (n = 14–93) from the different SWCNT-VDAC complexes evaluated. Equal letters indicate the absence of significant differences (p > 0.05) between the different single-walled carbon nanotubes. The dotted red line represents the average FEB value (−5.6 ± 1 Kcal/mol) determined for the adenosine triphosphate (ATP), a natural substrate of the VDAC channel from the three different species like VDAC1-*Homo sapiens* (PDB ID: 2JK4, Resolution 4.1 Å), VDAC1-*Mus musculus* (PDB ID:3EMN, Resolution 2.3 Å), VDAC2-*Danio rerio* (PDB ID:4BUM, Resolution 2.8 Å) used as control. In all cases, the results were obtained using docking simulations (see Methods for details).

The present work evaluated the relationships between all the semi-empirical geometric and physico-chemical SWCNT-nanodescriptors inspired by their periodic properties^41–46^ and FEB values from the different SWCNT-VDAC complexes formed. Herein, FEB-mean values of the SWCNT-VDAC complexes were significantly negative (p < 0.05) when compared with FEB-mean values of the ATP-VDAC complexes, following a higher binding stability for SWCNT-VDAC complexes formed like FEB **(**SWCNT-VDAC2-*Danio rerio* complexes) > FEB (SWCNT-VDAC1-*Mus musculus* complexes) > FEB (SWCNT-VDAC1-*Homo sapiens* complexes) in all cases. Nevertheless, higher values of oxidized SWCNT interactions for FEB (SWCNT-COOH > SWCNT-OH) were found in VDAC2-*Danio rerio* when compared to VDAC1-*Homo sapiens* and VDAC1-*Mus musculus* (p < 0.05) (Fig. 1 **)**.

**Figure 2:**
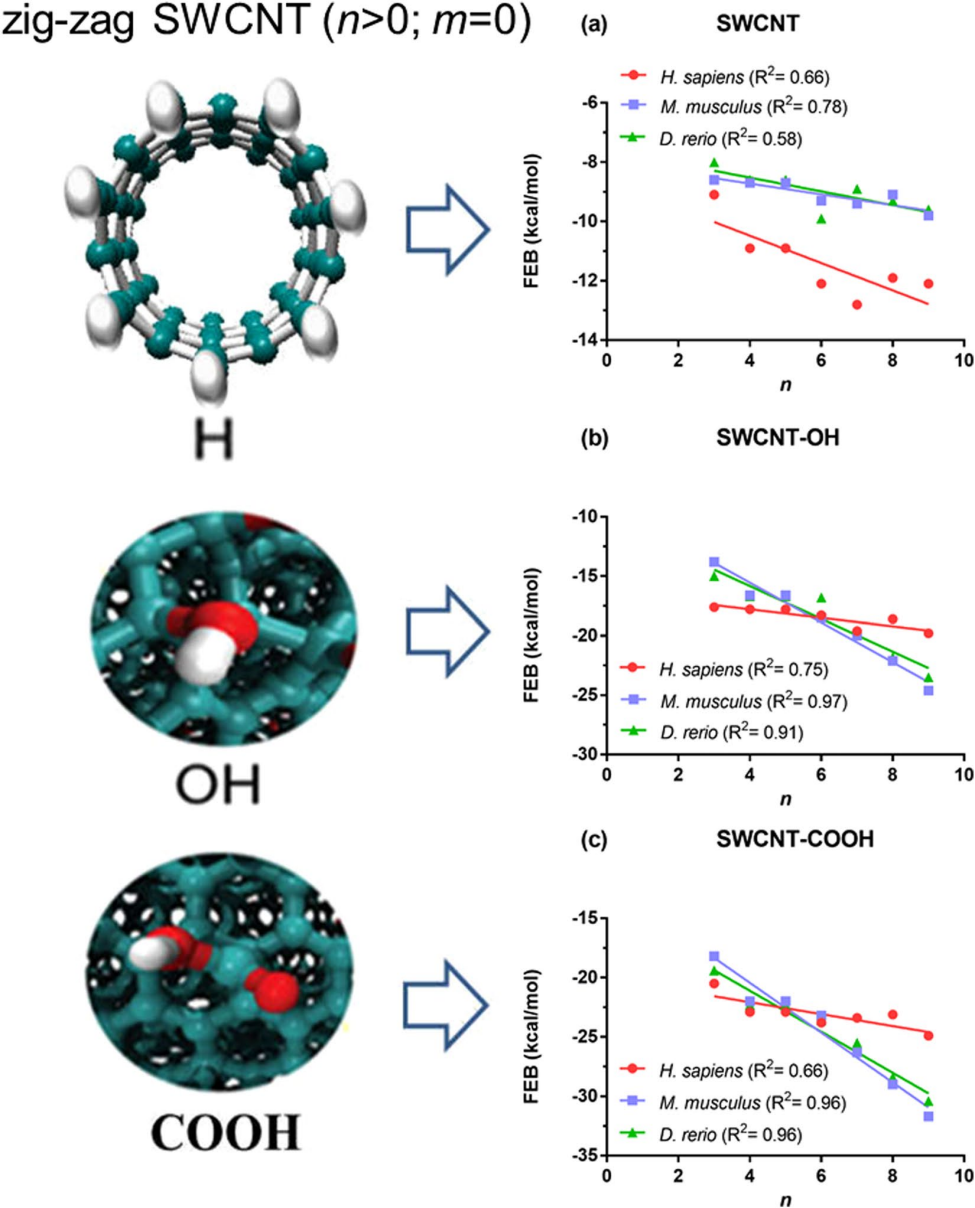
Linear relationships between free energy binding (FEB, in kcal mol^−1^) and the *n*-Hamada index for zigzag single-walled carbon nanotubes (*n* > 0, *m* = 0) regarding the VDAC channels from different species (mouse: *Mus musculus*, zebrafish*: Danio rerio*, human*: Homo sapiens*). (**a**) Pristine carbon nanotubes (SWCNT), (**b**) hydroxylated carbon nanotubes (SWCNT-OH), (**c**) carboxylated carbon nanotubes (SWCNTs-COOH). For all relationships, the determination coefficient (R^2^) is included.

According to the evidence obtained by Hiller *et al*.^47^ the VDAC channel diameter of wall-to-wall is ~34–38 Å, the maximum dimension limits being those allowing SWCNT-interaction with the VDAC-binding active site. Indeed, the assayed SWCNT diameters oscillated from 2.35 to 12.21 Å in the DS and, in all cases, the length was 10 Å, meaning that under the *in silico* conditions employed here, there were no steric constraints for the SWCNT-VDAC interaction with the VDAC-catalytic site. However, in general, a high proximity of the SWCNT family members with the voltage-sensing N-terminal α segment was observed, which is interesting because it explains the toxicodynamic mechanisms based on SWCNT properties and interatomic distances to key VDAC-functional and regulatory residues. The oxidized SWCNT-moieties (COO^−^ and OH) could be relevant for electrostatic interactions with the VDAC hydrophobic binding active site formed by cationic residues (arginine and lysine) closely involved in the each of the phases of mitochondrial ATP-transportation^1^. Moreover, the FEB docking results obtained for the electro-topological SWCNT-properties (diameter-chiral topology and functionalization) and the VDAC-affinity relationship suggest that the Hamada index *n* is a valuable SWCNT nanodescriptor, able to predict the SWCNT-VDAC interactions in zigzag SWCNT topologies (*n* > 0; *m* = 0) based on the determination coefficient (R^2^). In this regard, a significant linear correlation (0.66 ≤ R^2^ ≤ 0.97; p < 0.05) was observed between *n* and FEB for all zigzag SWCNT topologies. However, theR^2^ between *n* and FEB varied for different CNT-types, following the order: SWCNT-COOH > SWCNT-OH > SWCNT (Fig. 2). Particularly, the affinity of zigzag SWCNT based on R^2^ takes high values according to VDAC-interspecies criteria following the order VDAC1-*Mus musculus* (0.78 ≤ R^2^ ≤ 0.96), VDAC2-*Danio rerio* (0.58 ≤ R^2^ ≤ 0.96) and VDAC1-*Homo sapiens* (0.66 ≤ R^2^ ≤ 0.75).

On the other hand, the *m*-Hamada index does not seem to be an important nanodescriptor of SWCNT-VDAC interactions, since a lack of significant correlation (p > 0.05) with the FEB values was observed for the three SWCNT-topologies (zigzag, armchair, and chiral), for all the SWCNT-functional groups (H, OH, and COOH) considered and considering the VDAC-channels from different species see Figshare^48^ (Fig. SM01).

**Figure 3:**
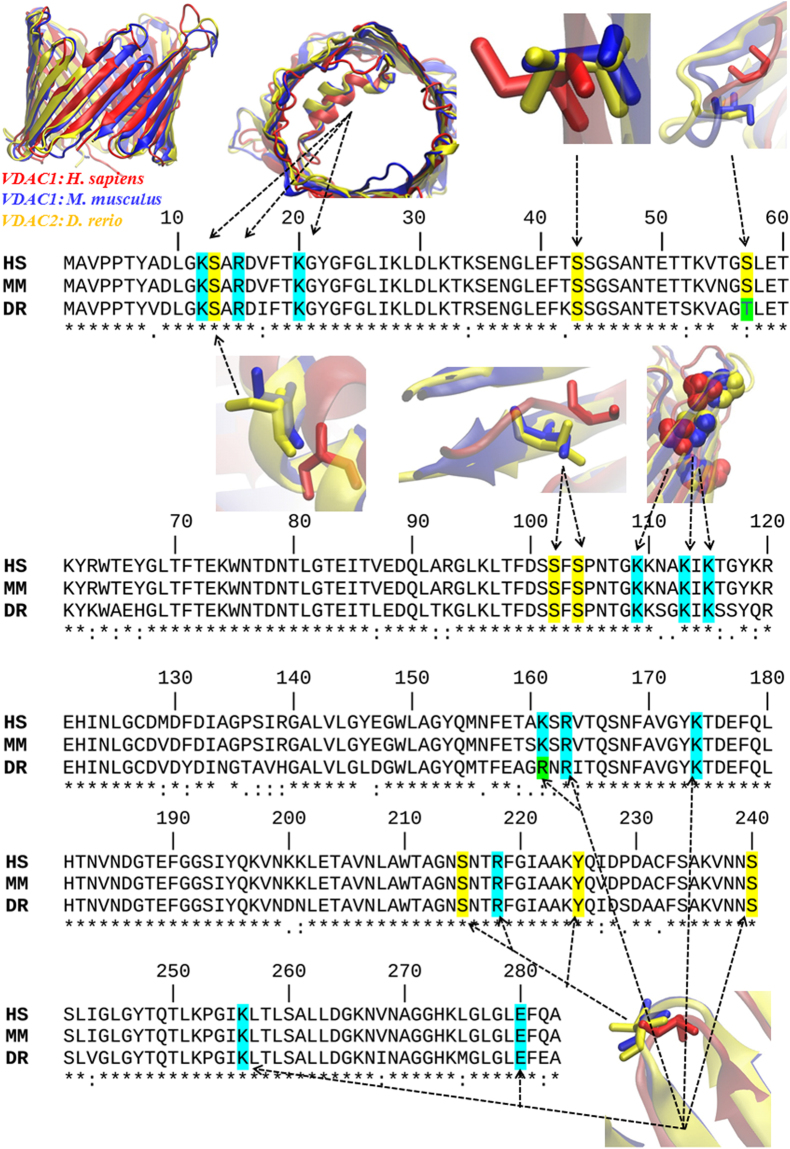
Primary sequence alignment with the position of VDAC-active binding site residues for the ATP transport. The figure includes details of 3D VDAC alignment of amino acid residues known to be phosphorylated (in yellow) (REF) and of the ATP transport (light blue). Note the high homology for VDAC critical residues both for ATP transport and phosphorylation. The alignment was performed for the three analyzed species: VDAC1-*Homo sapiens* (HS), VDAC1-*Mus musculus* (MM) and VDAC2-*Danio rerio* (DR). Note that in the case of residue lysine (K161), it is replaced by the basic residue arginine (R) with a similar ability to that of ATP transport, and the regulatory residue serine (S57) is replaced by tyrosine (T57) with the same ability to be phosphorylated in VDAC2-*D*. *rerio* (light green).

VDAC-active binding site residues (Arg 15, Arg 163, Arg 218, Lys 12, Lys 20, Lys 109, Lys 113, Lys 115, Lys 161, Lys174, Lys 256) that are involved in the ATP-transport have been shown to be fully-conserved in all the species considered (Fig. 3).

In this sense, a “common binding site substrate” can be proposed for ATP-VDAC interaction in the same biophysical environment to that where the SWCNT-family members were tested by DS with VDAC channel. This would support extrapolation criteria of the potential VDAC-nanotoxicity induced by SWCNT (SWCNT-H, SWCNT-OH, SWCNT-COOH) from any species assayed to other eukaryotic species not tested, independently of their phylogenetic location.

**Figure 4:**
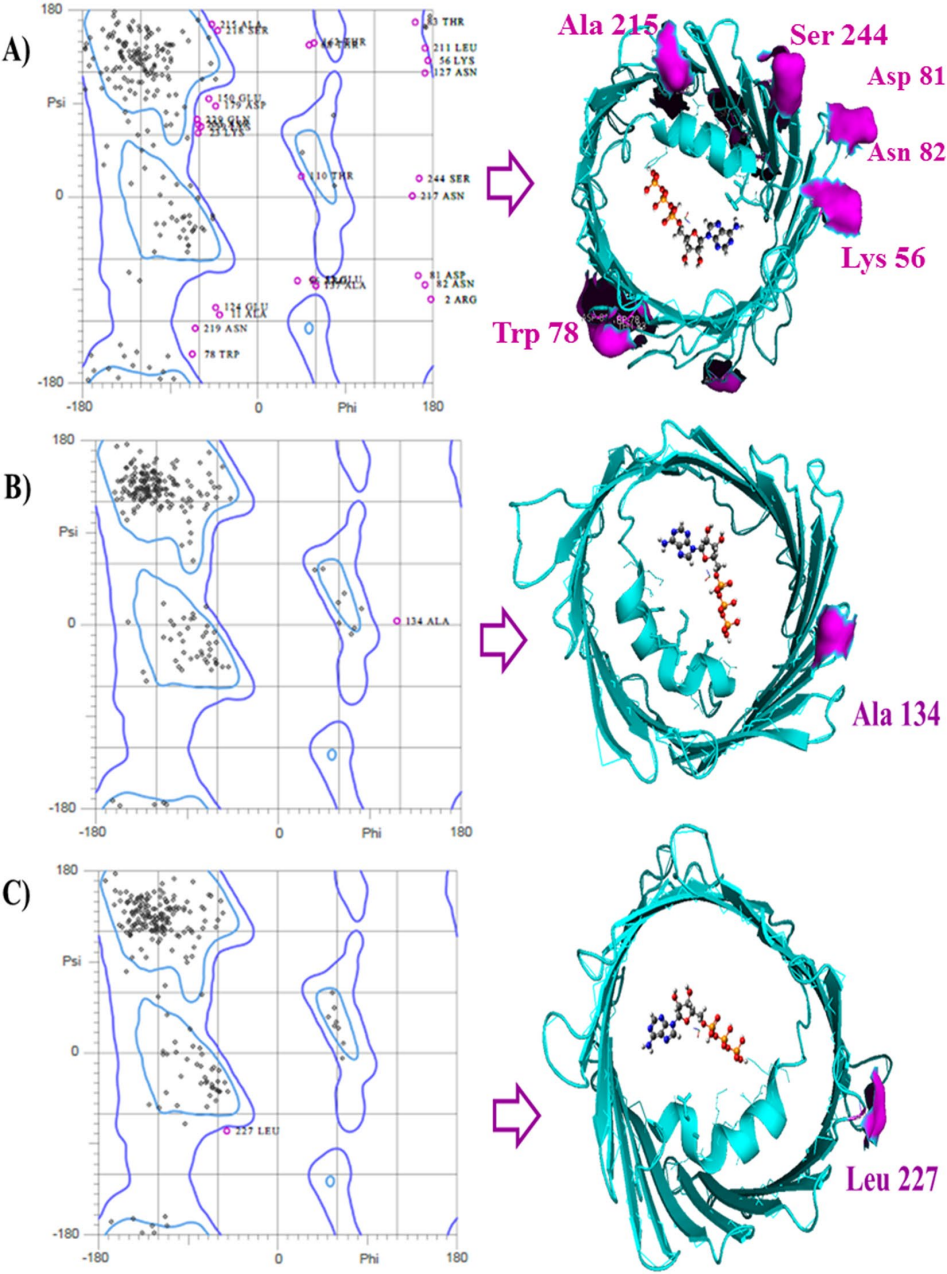
Representation of Ramachandran diagrams (general cases) (left) and spatial distribution of Ramachandran outliers in each VDAC.pdb x-ray structure analyzed (right). Figure shows the similar patterns of the VDAC geometry based on all the possible combinations of dihedral angles of torsion Ψ (Psi) versus Φ (Phi) of the each amino acid residues of VDAC of the different species analyzed: VDAC1-*Homo sapiens* PDB ID: 2JK4 resolution 4.1 Å (**A**); VDAC1-*Mus musculus* PDB ID:3EMN resolution 2.3 Å (**B**), and VDAC2-*Danio rerio* PDB ID:4BUM resolution 2.8 Å (**C**). The stereochemical spatial distribution of the Ramachandran outlier residues (marked in violet) in each VDAC.pdb x-ray structure analyzed (right) confirms that these residues do not belong to the ATP-VDAC active binding site of the VDAC pore domain of the voltage-sensing N-terminal α-helix on the polypeptide formed by the basic residues (Lys12, Lys20, Lys109, Lys113, Lys115, Lys161, Lys174, Lys256).

It is important to note that to avoid obtaining false positives on flexible-docking interactions (SWCNT-VDAC-channels) it was verified the absence of restricted-flexibility for each VDAC-residues from the three VDAC.pdb x-ray structures (PDB ID: 3EMN, 4BUM, and 2JK4) through Ramachandran plots. All the possible conformations of each VDAC-residue is defined by the torsion values of the dihedral angles (Psi) and (Phi) around the C_alfa_ of the peptide-bond of the VDAC-residues and can be represented by Ramachandran plot. For this instance, allowed torsion values of Ψ (Psi) versus Φ (Phi) of a given VDAC-residue are found within the contour lines of the Ramachandran plot (Ramachandran favored). Otherwise, is considered as disallowed and the torsion values of dihedral angles Ψ (psi) versus Φ (phi) appear outside of the Ramachandran contour line (Ramachandran outliers). Note that the VDAC-active binding site residues (Arg15, Arg163, Arg218, Lys12, Lys20, Lys109, Lys113, Lys115, Lys161, Lys174, Lys256) of the VDAC-cavity domain of the voltage-sensing N-terminal α-helix and also for VDAC- phosphorylation binding site regulatory residues (Ser13, Ser43, Ser102, Ser104, Ser240, Ser57, Ser215, Tyr225) considered as flexible in the docking study were not identified as Ramachandran outliers^49–51^ (Fig. 4).

It should be pointed out that the identified Ramachandran outliers were not key residues for ATP-transport and phosphorylation (showed in Fig. 3)^49–51^.

**Figure 5:**
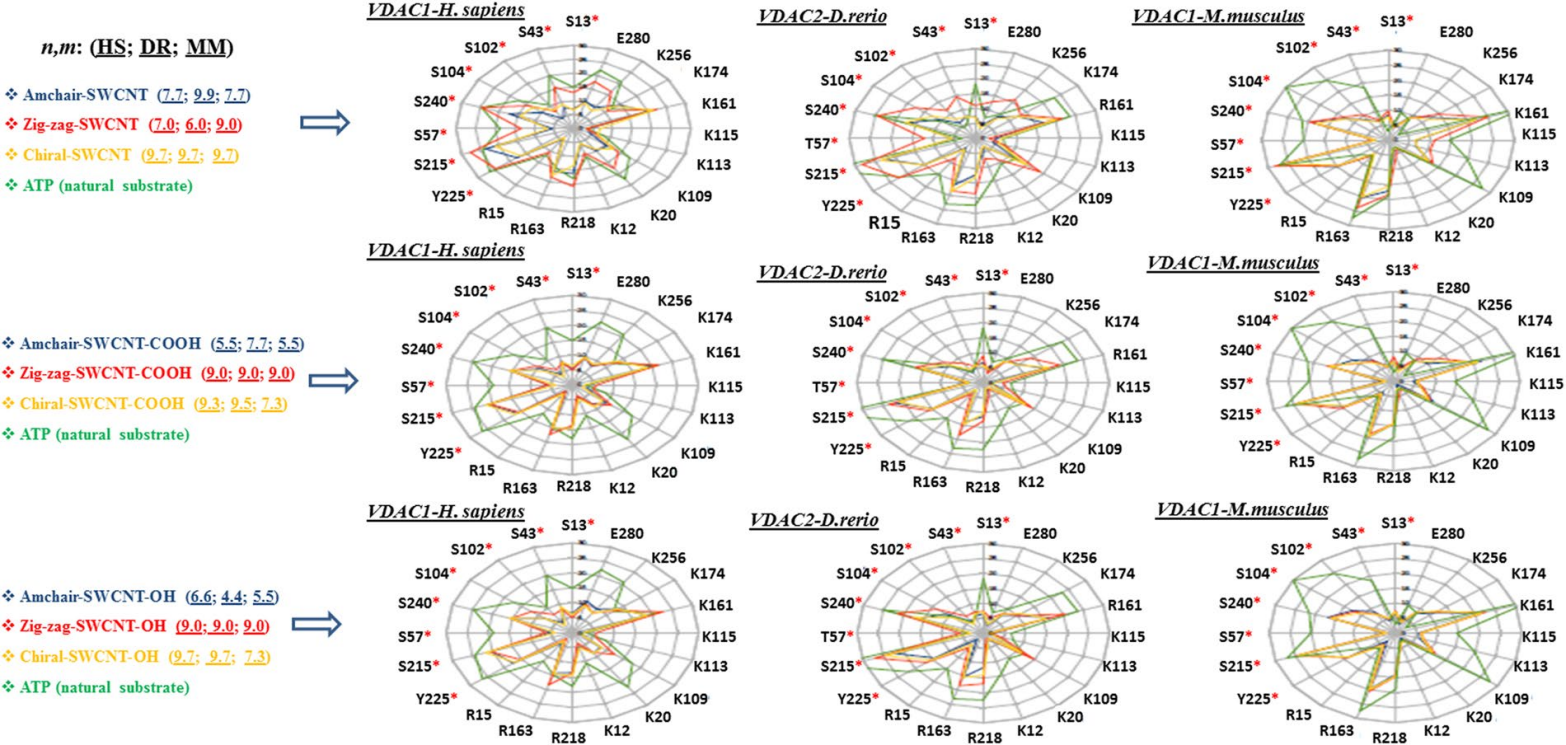
Radar plots showing the relative interatomic distances (scale: 0, 5, 10, 15, 20, 25, and 30 Å) of SWCNT family member (pristine-SWCNT, SWCNT-OH, SWCNT-COOH) with different *n*, *m*-Hamada index for the most negative FEB value results based on the final position after SWCNT-VDAC docking simulations for the three VDAC channels (HS: *Homo sapiens*; DR: *Danio rerio*; MM: *Mus musculus*). The amino acid residues of ATP-VDAC active binding site are marked with R for arginine (R15, R163, R218); K for lysine (K12, K20, K109, K113, K115, K161, K174, K256) and E for glutamate (E280). Red asterisks (*) show VDAC-phosphorylation binding site experimentally determined residues^9^: *S code for serine (*S13, *S43, *S102, *S104, *S240, *S57, *S215) and *Y code for tyrosine (*Y225). Radar plots show that the relative interatomic distances (Å) for SWCNT members with different geometric configurations such as armchair (blue line), zigzag (red line), chiral (orange line) and different types of SWCNT oxidation (OH, COOH) are similar and, in some cases, lower than ATP-relative interatomic distances (green line) used as control.

*In silico* results for the Euclidean SWCNT-interatomic distance values (**Å**) relative to ATP-VDAC active binding site residues used R code letter for arginine (R15, R163, R218), K code letter for lysine (K12, K20, K109, K113, K115, K161, K174, K256) and E code letter for glutamate (E280). For most of the SWCNT analyzed, the inter atomic distances for the SWCNT-VDAC interaction with the amino acid residues cited above were very similar or, in some cases, lower than the interatomic distance of these residues for the ATP-VDAC interaction (Fig. 5).

**Figure 6:**
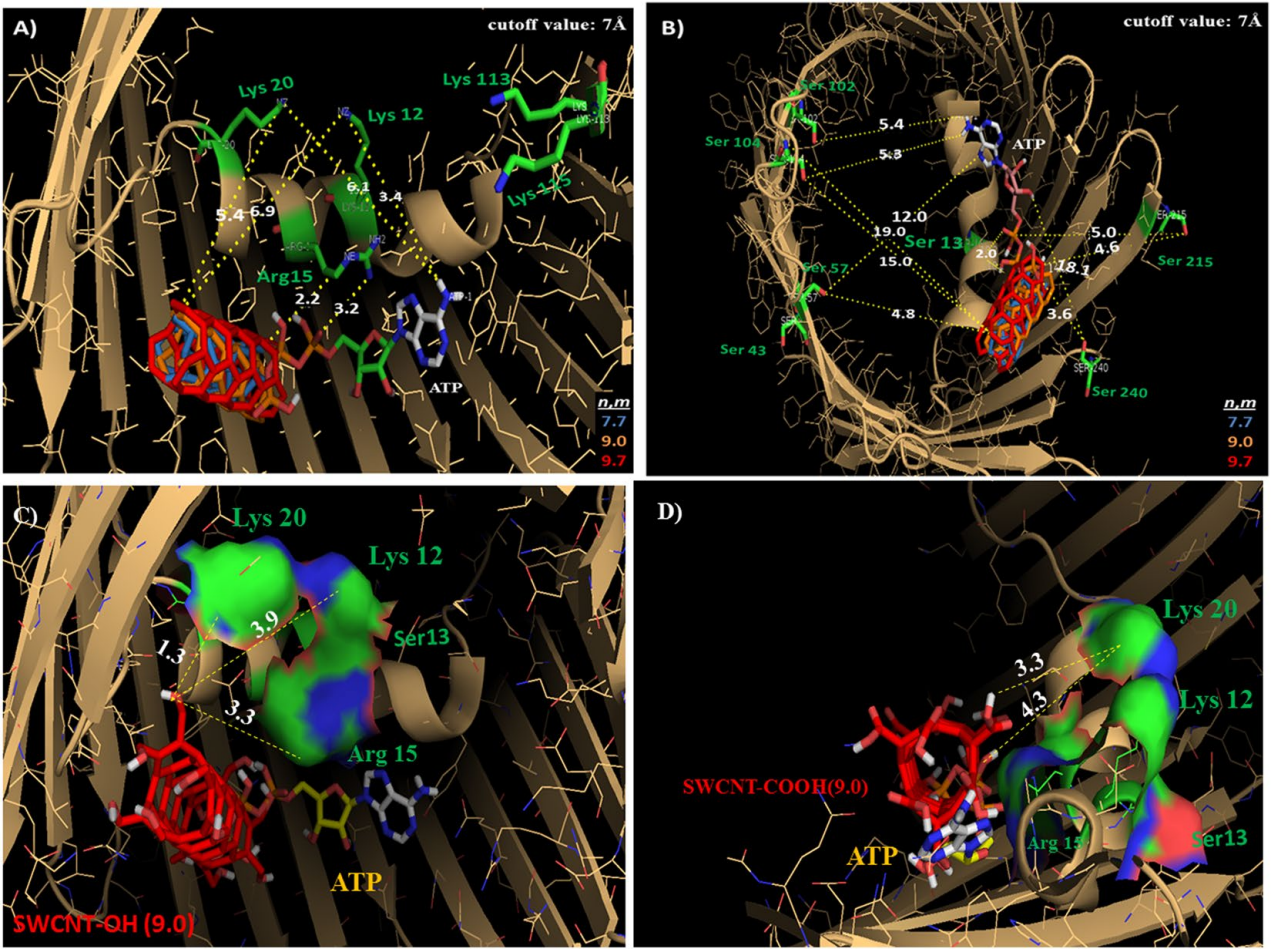
(**A**) Cartoon representation of the docking results in VDAC1-*Mus musculus* showing the calculated lower interatomic distances (d_ij_ cutoff value ≤ 7 Å) represented by yellow dashed lines for overlapping pristine-SWCNT with different *n*,*m*-Hamada indexes such as SWCNT (7.7) (blue), SWCNT (9.0) (orange), SWCNT (9.7) (red) compared to ATP (natural substrate of VDAC) from the supercritical ATP-VDAC active binding site residues (Lys 20; Lys 12; Arg 15). (**B**) Supercritical VDAC phosphorylation binding site regulatory residue (Ser 13) located in the VDAC pore domain of the voltage-sensing N-terminal α-helix show the calculated lower interatomic distances (d_ij_ < 2 Å) for overlapping pristine-SWCNT with different *n*,*m*-Hamada indexes such as SWCNT (7.7) (blue), SWCNT (9.0) (orange), SWCNT (9.7) (red) compared to ATP. (**C** and **D**) The calculated lower interatomic distances (d_ij_ ≤ 5 Å) from the supercritical ATP-VDAC active binding site residues (Lys 20; Lys 12; Arg 15) and also from the phosphorylation regulatory residue (Ser 13) on zigzag oxidized SWCNT members, such as SWCNT-OH (9.0) and SWCNT-COOH (9.0) compared to ATP in the VDAC voltage sensing pore domain, depicted as van der Waals surface (red is acidic, blue is basic). All docking images were designed using open-source Pymol 1.7.x. This pattern is representative of the remaining cases, as shown previously in the radar plots.

The atoms-proximity (C, O, H atoms) for each SWCNT-type to key binding residue atoms of VDAC-channels (for the ATP-transport and VDAC-phosphorylation binding site residues) could offer relevant information about VDAC physiological function. Interatomic distances lower than 7 Å imply a high probability of interaction in terms of free energy of binding and low probability of obtaining false contacts^52,53^ (Fig. 6).

Considering the docking results the SWCNT-VDAC-interactions (VDAC-channel nanotoxicity) it should be reasonable to hypothesize that the deprotonated SWCNT-COO^−^ moiety of SWCNT-COOH should disrupt the association of the ATP^4−^ anion (phosphate tail) with the positively charged ε-amino group (N^+^ primary amines) of Arg15 or Lys residues (Lys12, Lys20) present at the bottom of the VDAC cavity in the three species studied by forming stable complexes of salt bridges.

Moreover, the presence of pristine SWCNT and oxidized SWCNT in the cavity of the VDAC pore could cause a partial or total narrowing in the VDAC-active site, and thus affect the VDAC conductance through weakness of the function of Lys256 and Glu280, which are associated with electrostatic stabilization of the ATP^4−^ with Arg 15-(N^+^) during the ATP passage through formation of hydrogen bonds in the voltage-sensing N-terminal α-helical segment. Potential SWCNT-interactions (H-bond) with Arg15 and other important positive residues, such as Lys113 and Lys115 in the VDAC-binding active site, could alter the rate of ATP-dissociation from the helix. This happens when the adenosine ring is released and ATP passes to Lys12, Lys20, and Lys109 on the N-terminal α-helix segment via Lys174 on the β-barrel wall, which is considered a limiting step in the ATP-efflux when the Euclidean SWCNT_i_-VDAC_j_ interatomic distance d_ij_ ≤ 7Å^52–54^. It is important to note that interatomic distance values relative to VDAC-phosphorylation binding site residues (Ser13) associated to regulation of ATP-efflux in VDAC-pore exhibit values of d_ij_ ≤ 5 Å for all the SWCNT-types tested.

Phosphorylation is an important mechanism involved in mitochondrial signaling pathways^9,55,56^. Then, these potential biochemical events could explain the difference observed for FEB-values (of SWCNT-VDAC complexes formed) and could still be viewed as a friend or a foe depending on the toxicological condition considered^57^ like the presence of zigzag SWCNT topologies with high *n* Hamada index. Besides, a recognized phenomenon of “edge effects”, which only appears in semiconducting zigzag SWCNT topologies, plays an important role in the mitochondrial channel nanotoxicity of zigzag SWCNTs^18^. The “edge effects” were not observed for metallic-armchair SWCNT, wherein the cycloparaphenylene aromatic system of the SWCNT-extremes were closed and without tips charge variation^18,58^.

### Perturbation Theory Modeling and Mechanistic interpretation

Herein, a PT-NQSBR approach for the prediction of SWCNT-VDAC docking free energy of binding (FEB values) was applied. The output of the PT-NQSBR model is the scoring function *f*(FEB)_query_ (or *f*(FEB_*ij*_)_calc_) of the FEB value for the query SWCNT. The scoring function *f*(FEB_*ij*_)_calc_ increases for higher values of probability of binding, with FEB < −5.6 kcal/mol as a cutoff value that represents the FEB value for the ATP-VDAC interaction calculated in this work (see Fig. 1). The best NQSBR model found is shown below, according the following equation (1):

1 $$\begin{array}{rcl}f{(FE{B}_{ij})}_{calc} & = & {\rm{0.044984}}\cdot f{(FE{B}_{ij})}_{{\rm{\exp }}ected}+{\rm{0.124831}}\cdot {V}_{01}\\  &  & +\,{\rm{0.386548}}\cdot {{\rm{V}}}_{{\rm{11}}}+{\rm{0.004531}}\cdot {{\rm{V}}}_{{\rm{14}}}-{\rm{0.519378}}\\ {N}_{{\rm{total}}} & = & {\rm{405}}\quad {\chi }^{{\rm{2}}}=206.47\quad p < {\rm{0.005}}\end{array}$$

This equation is able to discriminate between the SWCNTs that bind strongly to VDAC (FEB < −5.6 kcal/mol) and those with a weak binding site (FEB ≥ −5.6 kcal/mol), only by including the FEB-expected value (*f* (FEB_ij_)_*expected*_), *n*-Hamada index (V01), *C*_*h*_-Chiral vector (V11), and the semi-empirical radial breathing mode V_RBM_ (V14)_._ This LDA model has values of accuracy, specificity, and sensitivity in the range of 73.0–94.7% for the training series, and in the range of 78.0–98.0% for the external validation series (Table 1).

**Table 1:** Results of the LDA analysis using a PT-QSBR model that discriminates between SWCNT strong binding interactions (FEB < −5.6 kcal/mol) and SWCT weak interactions (FEB ≥ −5.6 kcal/mol) with VDAC.

Data subset	Statistical		Observed values	
Training	Parameter	%	(FEB ≥ −5.6)_calc_	(FEB < −5.6)_calc_
(FEB ≥ −5.6)_obs_	Specificity	73.0	111	41
(FEB < −5.6)_obs_	Sensitivity	94.7	8	144
Total	Accuracy	83.9		
Validation			(FEB ≥ −5.6)_calc_	(FEB < −5.6)_calc_
(FEB ≥ −5.6)_obs_	Specificity	78.0	39	11
(FEB < −5.6)_obs_	Sensitivity	98.0	1	50
Total	Accuracy	88.1		

The data in the table show the statistical parameters (Stat. Param.) for specificity, sensitivity, and accuracy for both the training dataset (for model estimation) and the validation dataset (for model evaluation). In each case, the numbers of cases correctly or incorrectly classified are mentioned in the table. FEB stands for Free Binding Energy.

These results obtained with the LDA-NQSBR model support the hypothesis of a linear relationship for the strength of SWCNT-VDAC interactions based on the input variables (SWCNT nanodescriptors) and the output variables *f*(FEB_*ij*_)_calc_ (Table 2).

**Table 2:** Expected values of free energy of binding (FEB, in kcal/mol) of SWCNT-VDAC interaction.

Species	SWCNT type	SWCNT function	Mitochondrial Channel	<FEB>
*Danio rerio*	Pristine	H	VDAC2	−10.20
	oxidized	COOH	VDAC2	−29.06
	oxidized	OH	VDAC2	−22.80
*Mus musculus*	Pristine	H	VDAC1	−10.44
	oxidized	COOH	VDAC1	−20.63
	oxidized	OH	VDAC1	−17.44
*Homo sapiens*	Pristine	H	VDAC1	−12.87
	oxidized	COOH	VDAC1	−5.44
	oxidized	OH	VDAC1	−17.11

**Figure 7:**
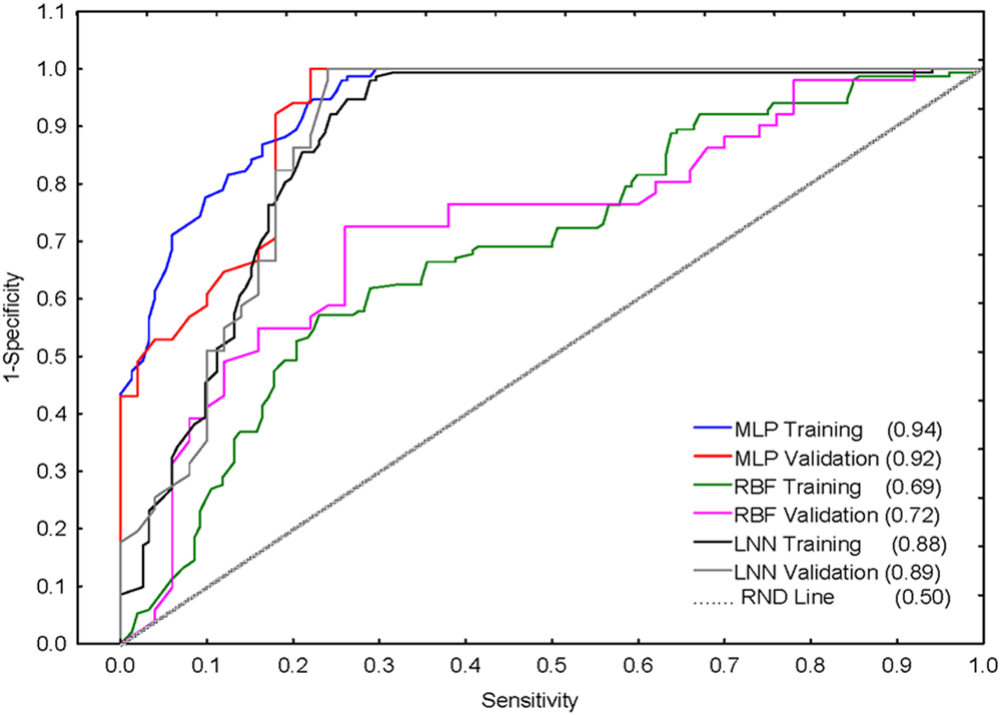
Results of the ROC curve analysis. Training and validation sets of the statistical parameters are represented by the area under receiver operating characteristic curve (AUROC) according to multiple layer perceptron (MLP), radial basis function (RBF) and linear neural network (LNN). The AUROC values are presented in brackets.

However, both linear and non-linear Artificial Neural Networks (ANN) analyses were carried out to calculate the Receiver Operating Characteristic (ROC) curves and obtain more evidence to support the linear hypothesis of SWCNT-VDAC interactions. The ROC analysis may also contribute to discard more extreme alternatives, such as the non-linear hypothesis or the random classification^59^, due to the large number of SWCNT-nanodescriptors used. In fact, the ROC graph shows high values of the area under the ROC curve (AUROC) of ≈0.9 in training and validation series for the Linear Neural Network (LNN) model with the same input variables used in the LDA NQSBR model (Fig. 7).

These values are notably higher than the expected values of AUROC = 0.5, typical of a random classification. The non-linear model corresponding to a Multiple Layer Perceptron (MLP) has also a very high AUROC = 0.92 to 0.94, but the values of accuracy, specificity, and sensitivity (in the range of 80.9–84.2%) are not higher than those for the LDA and LNN linear models. Finally, the Radial Basis Function (RBF) topology shows also notably lower AUROC ≈ 0.7 values in the training and validation datasets. Overall, the ROC curve analysis supports our selection of a linear hypothesis to predict the SWCNT-VDAC interaction represented by the LDA model. *Vide infra* in the Materials and Methods section. To verify the results obtained with the LDA classification NQSBR model from the external dataset, the empirical physico-chemical parameter of pristine SWCNT was used, not tested in our original dataset obtained by Bachilo (2002)^44^.

**Figure 8:**
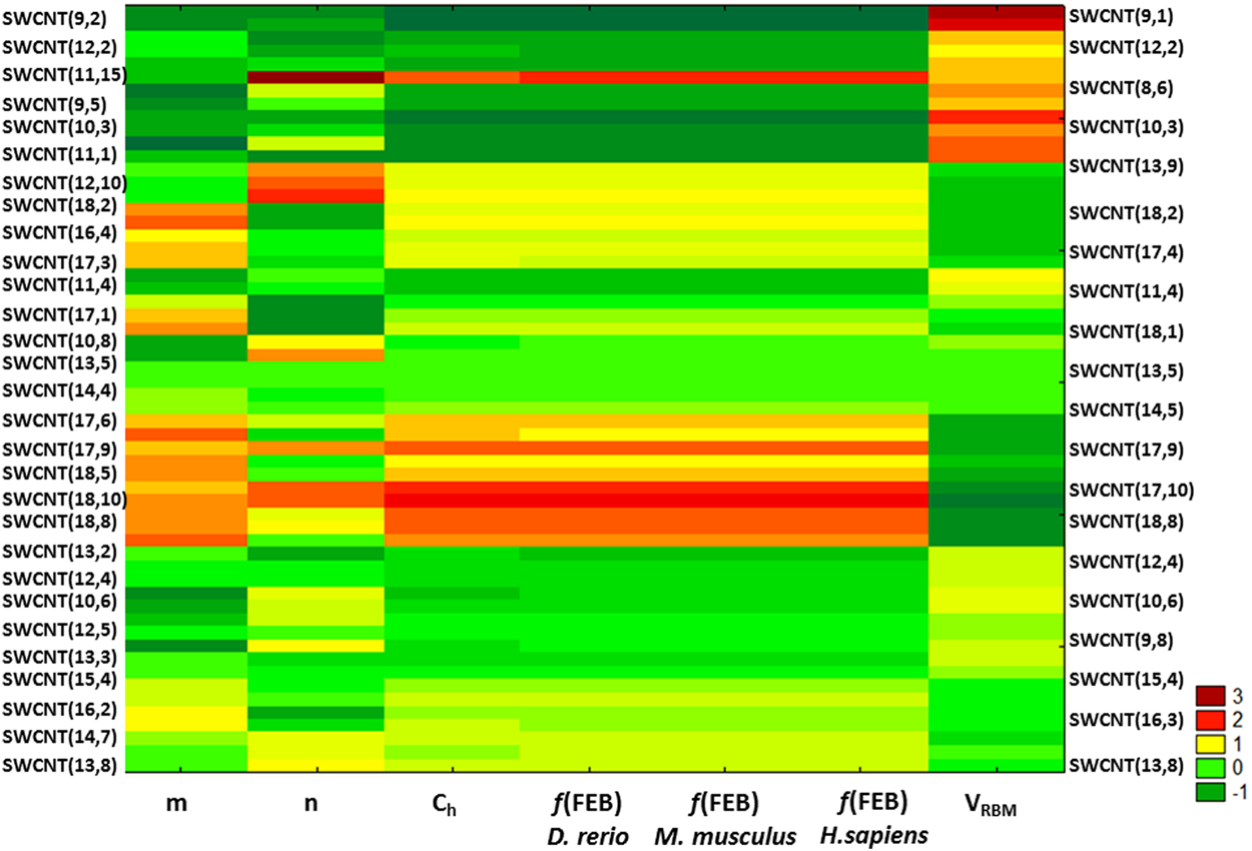
Two Way Joining Cluster (TWJC) analyses for the validation prediction of VDAC nanotoxicity of pristine SWCNTs using an external dataset (SM02.xlsx)^44,60^ by applying the obtained LDA classification NQSBR model. Each y-axis describes a vector of the elements corresponding to pristine SWCNT (y_i_) in individual layers. x-axis represents the main clusters of parameters from x_n_ row vectors used to predict the VDAC channel nanotoxicity for individual pristine SWCNT (x_i_), such as *n*: Hamada index, Ch: Chiral vector, V_RBM_: Radial Breathing Mode (cm^−1^) and expected *f*(FEB), as predictive function of free energy of binding used to classify strong and weak SWCNT-VDAC interactions in different species. The *m*-Hamada index was included in the TWJC analysis, but no correlation was observed in the performed NQSBR model for this SWCNT geometric parameter. Code numbers for SWCNT-VDAC interactions: very strong = 3; strong = 2; moderate = 1; and weak = −1. The results for the three species (zebrafish, *D*. *rerio*; mouse, *M*. *musculus*; and human, *H*. *sapiens*) are shown.

The multiple factors involved in VDAC channel nanotoxicity were represented based on the SWCNT-nanodescriptors using Two Way Joining Cluster (TWJC) (Fig. 8) from the external dataset available in Figshare^48^ (Table SM02).

A detailed analysis of the two-way contingency table with heat map contributes to verify the VDAC-affinity levels between similar clusters of relevant SWCNT-nanodescriptors. Particularly, the cluster obtained from x_n_ row vectors for *n*-Hamada index, Ch-Chiral vector, V_RBM_ and expected *f*(FEB) is consistent with the theoretical evidences obtained by the linear NQSBR model based on the classification power of strong and weak SWCNT-VDAC interactions, following the heat map intensity of the contingency table from pristine SWCNT (y_i_) in individual layers.

Futhermore, to explain SWCNT-VDAC interactions based on non-linear relationships classification and regression approaches using feature selection (FS) based on QSAR/QSPR non-linear algorithms were efficiently implemented. This procedure allowed eliminating redundant SWCNT-nanodescriptors that could cause a poor predictive potential and generalization of the results, as mentioned in the introduction of this work.

In this sense, J48 model could be used to filter SWCNT-nanodescriptors of new mitochondrial channel interactions based on V_RBM_ (V14), *n*-Hamada index (V01) and FEB-expected value see Figshare^48^ (Fig. SM03-A and Fig. SM03-B).

The results of this decision tree (J48 model) are consistent with our goal and have previously been verified in the linear NQSBR model. The V_RBM_ is a low frequency mode observed in the SWCNT-Raman spectrum linked to the CNT chiral-index assignments and it has a direct influence on the SWCNT-diameter (V06). Particularly, the *n*-Hamada chirality-index (V01) has a relevant meaning from the biophysical point of view to predict the SWCNT-VDAC interactions. This is because the *n*-Hamada index has a direct proportionality and strong linear relationship with the SWCNT-diameter (V06) following equation (2):

2 $${d}_{SWCNT(n,m)}=0.783\sqrt{{n}^{2}+nm+{m}^{2}}$$

Note that this relationship also applies for zig-zag topology SWCNT, where *m* = 0, which showed the most negative FEB-values (Kcal/mol). The average cavity-diameter wall-to-wall from the three VDAC-channel studied (VDAC1-*Homo sapiens*, VDAC1-*Mus musculus*, and VDAC2-*Danio rerio*) is about ~34–38 Å and these dimensions should the maximum to allow interaction with SWCNT. As mentioned previously this was in fact the case since the assayed SWCNT presented diameters raging from 2.35 to 12.21 Å.

We strongly suggest that SWCNT tested could cause partial or total distortions in the VDAC-active site affecting the ATP-efflux and/or modulate potential post-translational modifications in VDAC phosphorylation binding-site residues for large *n*-Hamada index (high SWCNT diameter) directly influencing the strength of interactions (more negatives FEB values) of the formed SWCNT-VDAC complexes available in Figshare^48^ (Fig. SM01).

The results based on the FS1 dataset are in general slightly better than the Pool dataset with all SWCNT nanodescriptors. Thus, Random Forest and K Star model were the best classifiers of SWCNT nanotoxicity on VDAC with the same TPR of 0.916, but AUROC of 0.978 and 0.968 see Figshare^48^ (Table SM04 and Table SM04-1).

FS3 dataset was used to decrease the dimensionality of the full dataset (Pool) by transforming the 19 dimensions based on SWCNT nanodescriptors into only 8 Principal Components to identify an optimal subset of SWCNT nanodescriptors that best map a relationship between structure and VDAC nanotoxicity see Figshare^48^ (Table SM04).

The Random Forest and Multilayer Perceptron are the best classifiers for SWCNT-VDAC interactions. The maximum AUROC/TPR was lower than FS0, FS1, FS2, with values of only 0.956/0.872. OneR model pointed to SWCNT molecular weight (V02) as an important SWCNT nanodescriptor see Figshare^48^ (Table SM04), SWCNT-molecular weight was not identified as a relevant SWCNT-nanodescriptor in the previous LDA NQSBR model. However, an increased SWCNT-molecular mass could be associated with a proportional increase in SWCNT diameter (V06). This affected the FEB values through potential steric collisions of the SWCNT-atoms with key binding site residues atoms near to N-terminal α-helical segment in the VDAC channels. Similarly, the FEBexp and SWCNT-molecular weight (V02) were used in FS4 dataset with a minimal dataset of SWCNT nanodescriptors. The Random Forest model demonstrated a similar performance by using FS1 and FS2 dataset of SWCNT nanodescriptors (AUROC = 0.978, TPR = 0.919). J48 classifier model was performed as a simple set of rules using only V02 and FEBexp with good performance for the predictive classification with AUROC of 0.897 and TPR of 0.889: SWCNT with molecular mass ≤ 2337 g/mol will have weak SWCNT-VDAC interactions, SWCNT with molecular mass = 2,337–6,245 g/mol and FEBexp ≤ −29.1 Kcal/mol will have strong SWCNT-VDAC interactions see Figshare^48^ (Fig. SM03-A and Fig. SM03-B).

In general, a better performance of the predictive classification model is obtained for the non-linear Random Forest model when all 19 SWCNT-nanodescriptors are used (Pool dataset) with an AUROC value of 0.973 and true positive rate (TPR) of 0.916. In this case, the MLP classifier (1 hidden layer with 19 neurons = number of SWCNT nanodescriptors (19) + no. of classes (2)) is better than the one used for the FS0 dataset (AUROC of 0.970 and TPR of 0.904) see Figshare^48^ (Table SM04 and Table SM04-1).

The last predictive test included additional categorical information, such as SWCNT Electroconductivity (metallic, semimetallic, semiconducting), SWCNT type (pristine SWCNT, oxidized SWCNT), SWCNT function (H, OH, COOH), VDAC isoform (VDAC1, VDAC2) and species (*Mus musculus*, *Homo sapiens* and *Danio rerio*) using Weka classifiers see details in Figshare^48^ (Table SM04 and Table SM04-1). Thus, by the addition of these categorical information to the Pool dataset with REPTree method, we obtained good prediction with AUROC/TPR/FPR = 0.926/0.889/0.111 (FPR = false positive rate). The classifier chooses only the information of FEBexp, *n*-Hamada index of the SWCNT (V01), SWCNT Molecular Weight (V02), SWCNT No. Bonds (V03), SWCNT Radio Rt (V05), SWCNT Nanotube diameter (V06), and species. Simple rules could be decrypted to predict the SWCNT-VDAC interaction from the plot: if V03 < 202 or if V03 ≥ 202 and V02 < 2,772 (g/mol) see Figshare^48^ (Fig. SM05-A). According to the species type, the SWCNT-VDAC interaction varies: for *Mus musculus* and *Danio rerio*, the SWCNT-VDAC interaction is strong, but in the case of *Homo sapiens*, there is a need for *n*-Hamada index and SWCNT Molecular Weight values to decide on the strength of interactions see Figshare^48^ (Fig. SM05-B). It should be noted that in the case of VDAC2-*Danio rerio*, the basic residue Lys161 is replaced by another basic residue (Arg161), with an equivalent physiological role in the ATP passage to cytosol^1^. The calculated interatomic distances relative to basic residues (Lys12, Lys20, Arg15, Arg163, Arg218, Lys109, Lys113, Lys115, Lys174, Lys256) for the SWCNT members with higher values of *n*-Hamada index and lower FEB values from pristine SWCNT and oxidized SWCNT are lower than 5 Å in most cases. The different single-walled carbon nanotubes followed the order SWCNT-COOH ~ SWCNT-OH < SWCNT, according to the interatomic distance values, as shown in the radar plot in Fig. 5, and considering a cutoff value of 7 Å.

To search for the best regression NQSBR model which predicts the FEB values, linear and non-linear methodologies were implemented by using the full dataset of nanodescriptors (Pool) as input. As an initial test, regression models were obtained by excluding the expected free energy of binding (FEBexp) to check the importance of the Perturbation Theory for this type of NQSBR models. All the methods provided regression models with very low performance (R^2^_test_ < 0.50). Thus, the importance of free energy of binding expected value (FEBexp) is confirmed for the prediction of new FEB values.

Finally, the best regression model that predicts FEB values for SWCNT-VDAC interactions based on the Pool dataset (all 19 SWCNT nanodescriptors) was provided by non-linear regression Random Forest models with R^2^_test_ = 0.833 and RMSE_test_ = 0.0844. Besides, NN provided a performance of R^2^_test_/RMSE_test_ = 0.757/0.0953. In addition, Random Forest Recursive Feature Elimination (RF-RFE) results were between RF and NN by selecting only 2 SWCNT nanodescriptors (9 out of 10 splits): FEBexp and V02 (the same SWCNT nanodescriptors used in the classification with the minimal dataset, FS4). With only two SWCNT-nanodescriptors, RF-RFE has a good performance to classify the SWCNT-VDAC nanotoxicity docking interactions, with R^2^_test_ of 0.798 and RMSE_test_ = 0.0953. If filters are applied in order to select optimal SWCNT nanodescriptors in RRegrs, the performance slightly decreases see Figshare^48^ (Table SM06 and Table SM06-1 **)**. However, the linear methods (LM and GLM) and the parametrical models (Lasso, PLS and ENET) show very low performance (R^2^_test_ < 0.50), whereas the applying of non-linear methods showed an increased R^2^_test_ over 0.75. See the pairwise model comparisons of R^2^_test_ (A) and RMSE_test_ (B) for multiple linear and non-linear regression models in Figshare^48^ (Fig. SM07).

## Conclusions

The Free Energy of Binding was determined using Molecular Docking Simulation with Virtual Screening Framework. The results showed that the affinity was statistically more negative for FEB values following the order **(**SWCNT-VDAC2-*Danio rerio*) > (SWCNT-VDAC1-*Mus musculus* > (SWCNT-VDAC1-*Homo sapiens*) ≈ (ATP-VDAC). In addition, the presence of zigzag topology and the COOH, OH functionalization are geometric, and toxicophoric SWCNT nanodescriptors are useful to describe their interactions with VDAC (order: COOH > OH > H). The FEB values of interactions based on these SWCNT nanodescriptors were shown to be stronger than the VDAC-natural substrate (ATP). However, some geometric and electronic properties, such as armchair and chiral configuration are uncorrelated with the VDAC affinity.

The broader theoretical importance of this work is not limited only to molecular docking simulation for solving nanotoxicological problems. The relevant alignments of sequences for the key functional and regulatory residues of VDAC channels from different species (represented as 3D-objects) should be relevant for the extrapolation of potential toxic effects of SWCNT to animal models, commonly used for nano-ecotoxicological studies on *Danio rerio*. Furthermore, classification and regression models able to predict strong and weak SWCNT-VDAC interactions (FEB values) were performed for the first time. Several decision trees were constructed to extract simple rules for the prediction of classes and exact values for the SWCNT-VDAC as channel nanotoxicity criteria linked to optimal SWCNT nanodescriptors, and involving interspecies comparison. The classification models demonstrated high accuracy, specificity, and sensitivity in training and validation series (73–98%) and area under receiver operating characteristic (AUROC) up to 0.978. The best regression method to predict the complex free binding energy was Random Forest with R^2^_test_ of 0.833 and RMSE_test_ of 0.0844. These *in silico* results pave the way for the rational designing of novel carbon nanomaterials with higher benefit-risk relationships, and for the creation of new regulatory decisions in nanotoxicology for the prediction/assessment of human health impact and environment nano-risks.

## Methods

### Docking simulation

To evaluate the interaction between the VDAC from three different species (VDAC1-*Mus musculus*, VDAC1-*Homo sapiens*, VDAC2-*Danio rerio*) and various types of SWCNT, a NQSBR flowchart was performed see Figshare^48^ (Fig. SM08). The first step consists of preparing the VDAC macromolecule structure files (receptor), which was obtained from the *RCSB Protein Data Bank* (PDB) x-ray structures^49^. Before the molecular docking, VDAC molecular structures were optimized using the AutoDock Tools 4 software for AutoDock Vina. The algorithm includes the removal of crystallographic water molecules and all the co-crystallized VDAC ligand molecules, such as Lauryl dimethyl-amine-N-oxide (LDA) or C_14_H_31_NO from VDAC2-*Danio rerio*.

Moreover, hydrogen atoms were added, according to appropriate hybridization geometry, to those atoms based on built-in modules to add partial charges, protonation states followed by bond orders assignment and set up rotatable bonds of the different VDAC channels.pdb x-ray structures.

In the second step, the SWCNT ligands (pristine SWCNT or SWCNT-H) structures were carefully modeled taking into account general SWCNT nanodescriptors such as semi-empirical values for [n] and [m]-Hamada indexes, calculated by H. Yorikawa and S. Muramatsu in 1995, and other SWCNT parameters such as molecular weight, no. bonds, no. atoms, radio, diameter, hexagons number/1D unit cell, metallic and/or semiconducting; SC)^42–45^. Herein, the software Nanotube Modeler (http://jcrystal.com/products/wincnt/) version 1.7.5, registered by one of the authors (J.M. Monserrat), was used. Some SWCNT-H structures were oxidized either with carboxyl (-COOH) or hydroxyl (-OH) moieties using an advanced semantic chemical editor, Avogrado (Version 1.1.1 free software). The minimization of all the SWCNT ligands was performed using the MOPAC extension for geometry optimization based on the AM1-Hamiltonian method.

An *in silico* framework was developed to configure the virtual screening (VS) experiments in order to evaluate the various parameters. This framework has a Web interface in which the user configures the docking experiment and obtains the respective Python script to automatically perform the VS steps. In the framework interface, the user provides information regarding the receptor files (VDAC channels) and the folder in which all the SWCNT structures are stored. To evaluate the SWCNT-VDAC *in silico* interactions, Autodock Vina flexible molecular docking was implemented, open source software developed by Trott & Olson (2010). The cationic cluster formed by ATP-VDAC-active binding site residues (Lys12, Lys20, Arg15, Arg163, Arg218, Lys109, Lys113, Lys115, Lys161, Lys174, Lys256, Glu280) and the VDAC phosphorylation binding site residues, experimentally determined by Martel & Brenner (2014)^9^ (Ser13, Ser43, Ser102, Ser104, Ser240, Ser57, Ser215, Tyr225), obtained from *Homo sapiens-VDAC*, *Mus musculus-VDAC* and *Danio rerio-VDAC* were tested as flexible residues and the ligands (SWCNTs) were considered a rigid molecules^61^. In this context, the SWCNT-VDAC complexes of free energy of binding (FEB) from different species were calculated based on the score function which approximates the standard chemical potentials (ΔG_bind_). The implemented ΔG scoring function combines the knowledge-based potential and empirical information obtained from experimental binding affinity measurements. The FEB optimization algorithm for SWCNT-VDAC complexes from the different species were implemented in Autodock Vina scoring function with default Amber force-field parameters. The FEB of SWCNT-VDAC complex optimization was performed with a gradient and efficient local optimization algorithm of the free energy of binding based on a quasi-Newton method, such as Broyden-Fletcher-Goldfarb-Shanno (BFGS). The algorithm is a succession of steps consisting of a mutation and a local optimization, with each step being accepted according to the Metropolis criterion. The components are the position and orientation of the SWCNT (as rigid molecules), as well as the torsions values of the VDAC-flexible residues. Conformational relaxation (flexible docking) favors a significant gain of enthalpy of SWCNT-VDAC complexes non-associated with SWCNT intra-molecular deformation or vibrational decrease within VDAC active sites. This theoretical procedure was performed for the receptor binding cavity using Cartesian coordinates for VDAC1-*Homo sapiens* grid box size, with dimensions of X = 20 Å, Y = 22 Å, Z = 20 Å and the VDAC1- *Homo sapiens* receptor grid box center X = 28.036 Å, Y = 0.361 Å, Z = 5.176 Å. Cartesian coordinates for VDAC1-*Mus musculus* grid box size, with the average dimensions of X = 22 Å, Y = 26 Å, Z = 22 Å and the VDAC1- *Mus musculus* grid box center X = 14.826 Å, Y = 32.707 Å, Z = 13.009 Å. Finally, the Cartesian coordinates for VDAC2-*Danio rerio* grid box size, with the average dimensions of X = 24 Å, Y = 24 Å, Z = 22 Å and the VDAC2-*Danio rerio* grid box center X = -14.894 Å, Y = 20.794 Å, Z = -9.617 Å were used to evaluate the SWCNT-VDAC interaction of the three species studied, considering the ATP biophysical environment (VDAC active site) as control to evaluate the SWCNT-VDAC affinity. Several runs starting from random conformations were performed, and the number of iterations in a run was adapted according to the problem complexity. An exhaustiveness option set to 50 (average accuracy) in each docking calculation was used^61^. Furthermore, it was verified whether a high increase of the exhaustiveness docking parameter of 100 increased the simulation time keeping the same FEB results.

The docking free energy of binding output results (or FEB values) is defined by ΔG_bind_ values (affinity) for all docked poses of the formed complexes (SWCNT-VDAC) and include the internal steric energy of a given ligands (SWCNT) which can be expressed as the sum of individual molecular mechanics terms of standard-chemical potentials like: van der Waals interactions (ΔG_vdW_), hydrogen bond (ΔG_H-bond_), electrostatic interactions (ΔG_electrost_), and intramolecular ligands interactions (ΔG_internal_) from empirically validated Autodock Vina scoring function based on default Amber force-field parameters.

In fact, the force field parameters were validated from experimental data which are molecular mechanics terms based on the scoring function to ligand-receptor conformation-dependent parameters (SWCNT_i_-VDAC_j_ inter-atomic interactions) and the ligand conformation-independent parameters (SWCNT_i_-_i′_ internal interaction). These mechanistic force-field parameters included in the Autodock Vina scoring function were validated on the basis of a strong linear correlation (scoring capability) of experimental binding-affinity data (K_d_, K_i_, and IC_50_-values) of the original crystallographic protein-ligand complexes (>16,000 complexes from >114,000 x-ray crystallographic structure). Besides, Autodock Vina scoring function considers optimal-linear free binding energy coefficients from experimentally determined chemical potentials (ΔG_internal_) of ligands (SWCNT). Following this idea, Autodock Vina scoring function has an optimal docking performance, which can be efficiently applied to multiple ligand-receptor affinity problems (as SWCNT-VDAC interactions). It is important to note that overall docking force field parameters are based on distance-dependent atom-pair interactions (d_ij_) according to the general thermodynamic equations represented below:

3 $$FE{B}_{dock}\approx {\rm{\Delta }}{G}_{bind}={\rm{\Delta }}{G}_{vdW}+{\rm{\Delta }}{G}_{H-bond}+{\rm{\Delta }}{G}_{electrost}+{\rm{\Delta }}{G}_{\mathrm{int}}$$

4 $$\begin{array}{rcl}FE{B}_{dock} & \approx  & {\rm{\Delta }}{G}_{bind}={\rm{\Delta }}{G}_{vdW}\sum _{SWCNT-VDAC}(\frac{{A}_{ij}}{{{d}_{ij}}^{12}}-\frac{{B}_{ij}}{{{d}_{ij}}^{6}})+{\rm{\Delta }}{G}_{H-bond}\\  &  & \sum _{SWCNT-VDAC}E(t)(\frac{{C}_{ij}}{{{d}_{ij}}^{12}}-\frac{{D}_{ij}}{{{d}_{ij}}^{10}})+{\rm{\Delta }}{G}_{elec}\sum _{SWCNT-VDAC}332.0\frac{{q}_{i}{q}_{j}}{\in ({d}_{ij}){d}_{ij}}\\  &  & +\,{\rm{\Delta }}{G}_{{int}ernal}\{\sum _{SWCNT}\frac{{A}_{ij}}{{{d}_{ij}}^{12}}-\frac{{B}_{ij}}{{{d}_{ij}}^{6}}+\sum _{SWCNT}E(t)\times (\frac{{C}_{ij}}{{{d}_{ij}}^{12}}-\frac{{D}_{ij}}{{{d}_{ij}}^{10}})\\  &  & +\sum _{SWCNT}332.0\frac{{q}_{i}{q}_{j}}{4{d}_{ij}{d}_{ij}}+\sum _{SWCNT}{\gamma }_{k}(1+\,\cos ({\varpi }_{k}{\theta }_{k}-{\theta }_{0k}))\end{array}$$

ΔG = −RT(ln K_i_), R (gas constant) is 1.98 cal*(mol*K)^−1^, and K_i_ represents the predicted inhibition constants at T = 298.15 K. The first term of a 12-6/Lennard-Jones potential (with 0.5 Å smoothing) describes the *van der Waals* interaction as A_ij_/d_ij_^12^ (attractive Guassian function) and B_ij_/d_ij_^6^ (repulsive or hyparabolic function) to represent a typical Lennard-Jones interactions (SWCNT-VDACs), provided the Gaussian term is negative and the parabolic positive, d_ij_ is the surface distance calculated as d_ij_ = r_ij_ − R_i_ − R_j_, where r_ij_ is the interatomic distance and R_i_ and R_j_ are the radii of the atoms in the pair of interaction of SWCNT_i_-VDAC_j_ atoms. The second term is the pair consisting of an H-bond donor and an H-bond acceptor as a directional 12–10 hydrogen-bonding potential term such as B_ij_/d_ij_^12^ and C_ij_/d_ij_^10^ (H-bonding potential with Goodford directionality), where *E*(*t*) is an angular weight factor which represents the directionality of the hydrogen bonds and d_ij_ follows the criteria mentioned above. The third term represents the Coulomb electrostatic potential stored in the formed complex (SWCNT-VDAC)_ij_ of *N* charges (*q*_i_, *q*_j_) of pairs of charged atoms of SWCNT_(i)_ and VDAC_(j)_. For this instance, appropriated Gasteiger partial atomic charges of the VDAC-channels (VDAC1-*Homo sapiens*, VDAC1-*Mus musculus*, VDAC2-*Danio rerio*) were assigned. Herein, d_ij_ is the interatomic distance between the point charges as the reference positions of interaction based on distance-dependent dielectric constant. In the present study Autodock Vina based on Amber force field was parameterized with default options for the SWCNT-data set (pristine-SWCNT, SWCNT-OH and SWCNT-COOH) by summing up individual molecular mechanic contributions like: SWCNT-intra-molecular contributions, SWCNT-aromaticity criterion and the set number of active torsions moving of each SWCNT-ligand following to general preparation procedures of ligand^61^. For this instance, the fourth term of the equation (3) as (ΔG_internal_) was used to validate the internal steric energy of each SWCNT-ligand including dispersion-repulsion energy and a torsional energy through the sum of the default Amber force field parameters (ligand conformation-independent parameters of the Autodock Vina scoring function).

By the other hand, the electrostatic components were considered and the SWCNT-partial atomic charges were properly assigned with the Gasteiger-Huckel algorithm using partial equalization of orbital electronegativities (PEOE) after the addition of polar and non-polar hydrogen atoms. These steps were empirically calibrated by default Amber force-field parameters. Furthermore, Autodock conformational search space for the ligands (SWCNT-structures) were experimentally-validated with Autodock default options which include a randomized large training dataset for ligands properties, 50 genetic algorithm runs, and 25 million evaluations in each, and also including all default structural-parameters to predict the best position and orientation of the ligand (SWCNT-docking capability) taking into consideration the coordinate systems of the receptor (VDAC-channels). It is important to note that, molecular docking dimensionality based on degree of freedom (DOF) of the each member of the SWCNT-data set (pristine-SWCNT, SWCNT-OH and SWCNT-COOH) like: SWCNT-atom position/translation (x_i_, y_i_, z_i_ = 3), SWCNT-atom orientation/quarternion (q(x_i_), q(y_i_), q(z_i_), q(w_i_) = 4), SWCNT-number of rotable bonds/torsion (tor_1_, tor_2_, …, tor_n_ = *N*_tor_) and SWCNT-total dimensionality (total DOF = 3 + 4 + n) not have a significant weight in the FEB_dock_ based on the very small intra-molecular contributions of force field parameters of the SWCNT-ligand which were considered as rigid-bodies^61^ and considering the aforementioned SWCNT-geometry optimization based on the ΔG_internal_ minimization of all the SWCNT-ligands used in the present study.

Docking was found as energetically unfavorable when a FEB for SWCNT-VDAC complex ≥ 0 kcal/mol (worst crystallographic pose) shows either an extremely low or complete absence of binding affinity according to repulsive interactions. Following this criterion, SWCNT conformations with the lowest Gibbs docking free energy of binding (FEB negatives value) were obtained. The best root-mean-square deviation (RMSD) was considered as a criterion of correct docking pose accuracy for atomic positions below 2 Å^52,53^. This is comparable to the best of several knowledge-based docking scoring functions according to the equation (5).

5 $$RMSD(pos{e}_{{i}_{swcnt}},pos{e}_{{j}_{vdac}})=\sqrt{\frac{\sum _{n}{(ato{m}_{({i}_{swcnt})}-ato{m}_{({j}_{vdac})})}^{2}}{n}}$$

The next step consists of analyzing the results obtained from the molecular docking with respect to the final free energy of binding - FEB for the SWCNT-VDAC complex of each experiment. The minimum distances (interatomic distances) were calculated between VDAC atoms of the key amino acids of the receptor (VDAC: VDAC1-*Mus musculus*, VDAC1-*Homo sapiens*, VDAC2-*Danio rerio*) and SWCNT atoms at the best docking crystallographic binding position for ligands (SWCNT family: pristine SWCNT, SWCNT-OH, SWCNT-COOH and their respective geometric configuration like amchair, chiral and zigzag). The only considered interatomic distances were related to VDAC active binding site residues (Lys12, Lys20, Arg15, Lys 109, Lys 113, Lys115, Lys 161, Arg163, Lys174, Arg 218, Lys256) involved in the ATP transport to compare with the ATP-VDAC interatomic distances as reference control of potential ATP efflux inhibition. In addition, we calculated the interatomic distances of SWCNT related to VDAC phosphorylation binding site residues, experimentally determined by Martel & Brenner (2014)^9^: *S code letters (Ser 13, Ser 43, Ser 102, Ser 104, Ser 240, Ser 57, Ser 215) and *Y code letter (Tyr 225) from VDAC1-*Homo sapiens*, VDAC1-*Mus musculus* and VDAC2-*Danio rerio*.

Next, the Euclidean distances (*d*_ij_: SWCNT-VDAC interatomic distance) were calculated from all the atoms in the SWCNT to all the atoms in the VDAC channel in each species under study. The xyz-coordinates of all atoms of a VDAC channel (*x*: VDAC, *y*: VDAC, *z*: VDAC) were taken as input, and the xyz-coordinates (*x*: SWCNT, *y*: SWCNT, *z*: SWCNT) of all atoms of a SWCNT were taken as output (or a minimum distance) between a SWCNT_*i*_ atom and a VDAC_j_ atom, according to the following equation (6):

6 $${d}_{ij}=\sqrt{{({x}_{(i)swcnt}-{x}_{(j)vdac})}^{2}+{({y}_{(i)swcnt}-{y}_{(j)vdac})}^{2}+{({z}_{(i)swcnt}-{z}_{(j)vdac})}^{2}}$$

To evaluate the SWCNT_i_-VDAC_j_ interaction, a cutoff value of 7 Å was used, i.e., all atoms with distances *d*_ij_ ≤ 7 Å were considered as interacting atoms according to standard docking studies^52,53^.

### Performed molecular docking

For the docking tests, the following were used: VDAC channels from VDAC1-*Homo sapiens* (PDB ID:2JK4, resolution 4.1 Å), VDAC1-*Mus musculus* (PDB ID:3EMN, resolution 2.3 Å), VDAC2-*Danio rerio* (PDB ID:4BUM, Resolution 2.8 Å), and a combination of SWCNT ligands, such as armchair-H, armchair-COOH and armchair-OH (Hamada index *n* = *m*; 21 nanotubes); chiral-H, chiral-COOH and chiral-OH (no reflection symmetry; 93 nanotubes); zigzag-H, zigzag- COOH and zigzag-OH (Hamada index *m* = 0, *n* > 0; 21 nanotubes) and the ATP ligand molecule as reference control (C_10_ H_16_ N_5_ O_13_ P_3_, ATP model, SDF format from PubChem CID: 5957). All docking simulations were performed using the default values for Autodock Vina parameters.

### Statistical analysis

Mean values of free energy binding (FEB) from the various single-walled carbon nanotubes were compared through two-way analysis of variance in which the factors were SWCNT functionalization (-H, -OH and -COOH) and SWCNT electrotopological properties (chiral, armchair and zigzag). Normality and variance homogeneity assumptions have been previously verified. Pairwise comparisons were performed using the Newman-Keuls *post-hoc* test. In all cases, type I error was set at 0.05 (α = 5%). Quantitative structure-binding affinity relationships were evaluated through the use of stepwise multiple regressions, considering the FEB values of the SWCNT (SWCNT-H, SWCNT-OH and SWCNT-COOH)-VDAC complexes obtained from the different species (*Danio rerio*; *Mus musculus*; *Homo sapiens*) as dependent variables and several semi-empirical SWCNT quantitative nanodescriptors (*n*, *m*-Hamada indexes, chiral angle, molecular weight, diameter, etc.) as independent variables.

### VDAC channel alignments

Sequences of VDAC-1 from human *Homo sapiens* (NP_003365.1), VDAC1 from mouse *Mus musculus* (NP_035824.1) and VDAC2 from zebrafish *Danio rerio* (NP_001001404.1) were obtained from Gene Bank database (http://www.ncbi.nlm.nih.gov/genbank/). The alignments were performed on-line using the free software ClustalW2 (http://www.ebi.ac.uk/Tools/msa/clustalw2/). In addition, 3D structural alignment of VDAC from the different species tested was performed by VMD - Visual Molecular Graphics Software. Next, the PDB model X-ray structure validation from the different VDAC channels was performed by Ramachandran plots using MolProbity^51^.

## Model construction

### Dataset

The dataset contains 19 features or SWCNT nanodescriptors (see Table 3) based on the SWCNT periodic properties^41–45^ such as *m*-Hamada index (V00), *n*-Hamada index (V01), molecular weight (V02), number of bonds (V03), number of atoms (V04), CNT radio Rt in nm (V05), nanotube diameter dt in nm (V06), highest common divisor of (n, m) (V07), highest common divisor of (2n + m, 2 m + n) (V08), translation vector T in Å (V09), semi-empirical HOMO-LUMO band gap Eg in eV (V10), chiral vector in nm (V11), chiral angle θ (V12), mod(n−m, 3) (V13), estimated VRBM in cm^−1^ (V14), First van Hove Singularity Optical Transitions peak E11 in eV (V15), Second van Hove Singularity Optical Transitions peak E22 in eV (V16), and number of hexagons/unit cell (N/2) (V17). In addition, the Docking Free Energy of Binding (*FEB expected*) for the SWCNT groups (SWCNT type and SWCNT function as H, OH, COOH) was added using Perturbation Theory (PT).

**Table 3:** Variables used as input for the NQSBR model.

SWCNT nanodescriptors (V_k_)	Symbol
FEB (ATP) (Kcal/mol)	−5.6
Tube Length (Å)	10
*m*-Hamada index	V00
*n*-Hamada index	V01
Molecular Weight (g/mol)	V02
No. Bonds	V03
No. Atoms	V04
Radio Rt (nm)	V05
Nanotube diameter: d_SWCNT (*n*; *m*)_	V06
Highest common divisor of (n, m)	V07
Highest common divisor of (2n + m, 2 m + n)	V08
Translation vector: T(Å)	V09
Semi-empirical Homo-Lumo Bandgap E_g_(eV)	V10
Chiral vector: C_h_ (nm)	V11
Chiral angle (θ)	V12
mod (n−m, 3)	V13
Semi-empirical V_RBM_ (cm^−1^)	V14
First van Hove Singularity Optical Transitions peak-[E_11_] (eV)	V15
Second van Hove Singularity Optical Transitions peak-[E_22_] (eV)	V16
No. hexagon/unit cell (N/2)	V17

Thus, 19 SWCNT nanodescriptors and 405 cases were used to build NQSBR classification and regression models that can predict whether the SWCNT-VDAC interactions are weak or strong, along with FEB values.

### Performed NQSBR model based on Docking Perturbation Theory

In this section, a Nano-Quantitative Structure-Binding Relationship (PT-NQSBR model) was developed. To this end, a new prospective docking scoring function is used to predict the strength of the binding b_ij_ between the i^th^ CNT and the j^th^-VDAC from three different species. The free energies of binding (FEB), calculated in molecular docking experiments as the measure of b_ij_, were also employed. b_ij_ = 1 was considered when FEB_ij_ < −5.6 kcal/mol (strong binding interaction), otherwise b_ij_ = 0 (weak binding interaction). The cut-off FEB value was set at −5.6 kcal/mol, which represents the FEB value for the molecular docking interaction of the natural ligand substrate ATP with VDAC (calculated in this work, *vide infra*).

The re-formulation of the QSAR/QSPR approach was described, based on the Perturbation Theory (PT) to develop a new type of PT-NQSBR model for prospective classification of single-walled carbon nanotubes associated with VDAC channel nano-mitotoxicity. The main assumption of QSAR/QSPR models in general is that similar molecules have similar properties. Consequently, small changes (“*perturbations*”) in the structural system should correlate linearly with small changes in the values of their properties (biological activities). PT-QSPR models are very useful for the study of complex molecular systems with simultaneous multiple experimental boundary conditions.

In this regard, the QSPR approach of the Perturbation Theory is a mathematical formalism that starts by knowing the exact solution of a problem (for instance a SWCNT physico-chemical property for VDAC interaction) and continues by adding corrections or “perturbations”, according to the variations of different experimental conditions in order to predict a solution to a related problem without a known exact solution^9,30,31^. In our previous studies, Moving Average (MA) has been used to measure the deviations of the different input variables in PT models for several molecular bio-systems^18–20,31^. The PT-NQSBR model proposed herein is an additive polynomial equation expressed as follows:

7 $$f{(FEB)}_{calc}={e}_{0}+{{\rm{a}}}_{0}\cdot \langle f{(FEB)}_{\exp ected}\rangle +\sum _{k=1}^{k=14}{a}_{k}\cdot {\rm{\Delta }}{V}_{k}$$

8 $$f{(FEB)}_{calc}={e}_{0}+{{\rm{a}}}_{0}\cdot \langle f{(FEB)}_{{\exp }ected}\rangle +\sum _{k=1}^{k=14}{a}_{k}\cdot [{}^{query}V_{k}-{}^{ref}V_{k}]$$

The first input term is the function *f*(FEB)_expected_ =  < FEB > , which is the average value of FEB for a specific VDAC channel of one species for all SWCNTs of the same class with the same function (H, OH, or COOH), and the same electronic properties (metallic, semimetallic, semiconductor). It means that < FEB > can be considered as the expected value of FEB for the interaction of a SWCNT of the same class as the new SWCNT with the target protein VDAC (assuming a normal distribution). The second class of terms such as Vk, are values of the structural parameters (or SWCNT nanodescriptors) of the new SWCNT (or query SWCNT). Last, the difference (∆V_k_ = ^query^V_k_ − ^ref^V_k_) quantifies the perturbations (changes, distortions, etc.) of the SWCNT nanodescriptors (^query^V_k_) of the new SWCNT compared to those of the original reference SWCNT (^ref^V_k_). Please refer to Table 1 for further details on the employed model.

The Linear Discriminant Analysis (LDA) forward-stepwise algorithms from the STATISTICA software were used to fit the values of the parameters (a0, ak, bk and e0). In the PT-NQSBR model, the output *f*(FEB)calc is a function of the value of FEB for the new SWCNT structure, which contains the SWCNT nanodescriptors (Hamada index *n* and *m*, diameter, molecular weight, number of atoms, radial breathing mode or V_RBM_ frequencies, etc).

### Performed classification NQSBR models

Complex models, such as NQSBR classification models, were constructed using two types of applications: STATISTICA and Weka based on Machine Learning. With STATISTICA, the following models were calculated: Linear Discriminant Analysis (LDA), Multilayer Perceptron (MLP) and Radial basis function network (RBF). Weka provided an attribute selection tool where the process was separated into two parts: 1) Attribute Evaluator Method, through which attribute subsets were assessed and **2)** Search Method, which allows searching for the space of possible subsets of features (SWCNT nanodescriptors)^36^.

Weka was used to search for models using other 12 non-linear methods grouped into classes of classifiers such as *bayes* (Bayes Network, Naïve Bayes), *functions* (Multilayer Perceptron - MLP), *lazy* (IBk, KStar or K*), *rules* (Decision Table, JRip, OneR, PART), and *trees* (J48, Random Forest, REP Tree). Thus, 14 Machine Learning^36^ methods were used to compare the performance of prediction for weak or strong SWCNT-VDAC interactions.

The FEB values were transformed in two classes of SWCNT-VDAC nano-interactions: strong and weak, considering a FEB cut-off value (based on the FEB value of ATP-VDAC interaction) of −5.6 Kcal/mol, as mentioned above.

With STATISTICA, the datasets were randomly split into two subsets: training and test validation sets (75% training, 25% validation). Using Weka, the default 10-fold cross-validation (CV) was used. The performance of the classification models was measured with accuracy, specificity, and sensitivity. With Weka data mining algorithms, several variations of the initial dataset of SWCNT nanodescriptors were generated using filter methods (or feature selection, FS) to verify the influence of a high or low number of SWCNT nanodescriptors in the NQSBR model. The group of SWCNT nanodescriptors is more important to obtain a better model in order to describe the SWCNT-VDAC interaction (channel nanotoxicity) such as:

- *FS0* represents the dataset with the same SWCNT nanodescriptors of the best predictive linear NQSBR model obtained with STATISTICA (4 SWCNT nanodescriptors: FEBexp, V01, V11, V14);
- *Pool* contains all the features (SWCNT nanodescriptors: FEBexp, V00–V17);
- *FS1* was obtained by feature selection with parameters, using Evaluator (*CfsSubsetEval*) to assess subsets of SWCNT nanodescriptors that highly correlate with the class value and have low correlation with each other. In addition, Search (*Best First*) was used for 6 SWCNT nanodescriptors: FEBexp, V02, V03, V04, V10, V12) with a best-first search strategy to navigate attribute subsets which reduce training time, overfitting, and improves accuracy.
- *FS2* was obtained by feature selection with Evaluator (*Correlation Attribute Eval*) following the principle that features (SWCNT nanodescriptors) are relevant if their values vary systematically with the membership category. Search (*Ranker*) was also used, based on 8 SWCNT nanodescriptors: FEBexp, V02, V03, V04, V09, V11, V12, V14);
- *FS3* was obtained by Weka data mining feature selection using Evaluator (*Principal Components*) and Search (*Ranker*) based on 8 SWCNT nanodescriptors); the Principal Component^62^ transformation was used to study the influence of dataset dimension reduction by encoding the information of 19 SWCNT nanodescriptors into only 8 linear combinations of them;
- *FS4* contains only 2 SWCNT nanodescriptors that have proven to be very important for the models (*FEB expected*, V02).

The performance of the Classification Machine Learning NQSBR models from Weka are characterized by 3 criteria: maximum AUROC^63^, maximum TP Rate (true positive rate), minimum FP Rate (false positive rate), commonly used in the field of chemoinformatics^36^ and currently in computational nanotoxicology. To visualize and verify the results obtained with the LDA classification NQSBR model from the external dataset, we used a set of empirical physico-chemical parameters of pristine SWCNT, not tested in our original dataset, obtained by Bachilo *et al*. (2002)^44^. In addition, to represent the multiple factors involved in VDAC channel nanotoxicity, a Two Way Joining Cluster (TWJC) analysis was performed through heat map of contingency table^64^.

### Performed regression NQSBR models

The Pool dataset was used with the RRegrs methodology^33,37^ taking into account all the SWCNT nanodescriptos (V00–V17) to find the best regression model that predicts FEB values for the SWCNT-VDAC interactions. The initial dataset was normalized using R scripts. RRegrs is an R integrated framework that provides ten linear and non-linear regression models. The following regression methods were tested: Multiple Linear regression (LM), Generalized Linear Model with Stepwise Feature Selection (GLM), Lasso regression (Lasso), Partial Least Squares Regression (PLS), Elastic Net regression (ENET), Neural Networks regression (NN), Random Forest (RF), and Random Forest Recursive Feature Elimination (RF-RFE)^33^. Standard RRegrs parameters and methodology were applied: dataset automatically devided by RRegrs using 10 splits (train and test subsets)^33^. The selection of the best regression models used R^2^_test_ (regression coefficient for test subset) and RMSE_test_ (root-mean-square errors for test subset) values Figshare^48^ (Fig. SM02). As an initial test, regression models were sought by excluding the FEBexp, checking the importance of the Perturbation Theory for these regression models when predicting the SWCNT-VDAC interactions.

## Electronic supplementary material

Supplementary Figure SM01

Supplementary Table SM02

Supplementary Figure SM03

Supplementary Table SM04

Supplementary Figure SM05

Supplementary Table SM06

Supplementary Figure SM07

Supplementary Figure SM08

Supplementary Table SM041

Supplementary Table SM061
