# Supplementary material for: Decrypting Strong and Weak Single-Walled Carbon Nanotubes Interactions with Mitochondrial Voltage-Dependent Anion Channels Using Molecular Docking and Perturbation Theory
Source: Sci Rep. 2017 Oct 16;7:13271. doi: 10.1038/s41598-017-13691-8 (PMC5643473; doi:10.1038/s41598-017-13691-8)
Supplement: Supplementary file 1 — Supplementary Figure SM01 [file 41598_2017_13691_MOESM1_ESM.docx]

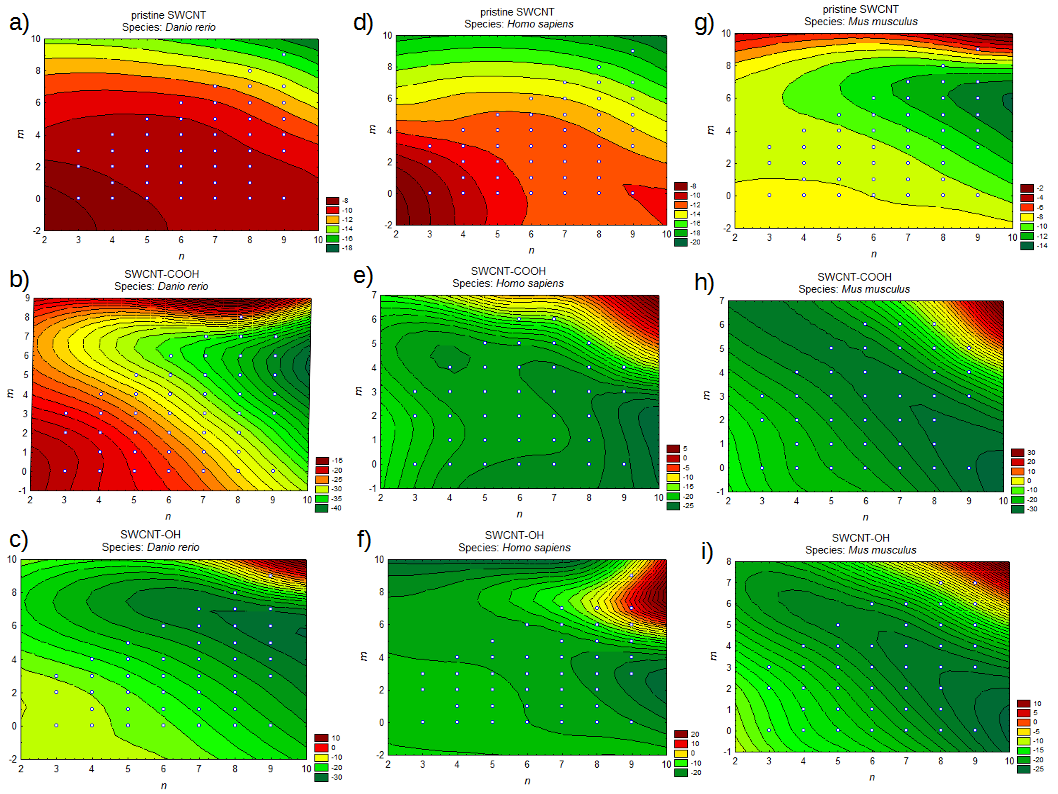


**SM01**. Two dimensional 2D-contour plot analysis of FEB values for all pristine and oxidized SWCNT-VDAC complexes as function of *n* and *m* chiral indices. Shows the SWCNT-VDAC2 complexes from *Danio rerio* (a, b and c)*,* SWCNT-VDAC1 complexes from *Homo sapiens* (d, e and f), SWCNT-VDAC1 complexes from *Mus musculus* (g, h and i).
