# Supplementary material for: Decrypting Strong and Weak Single-Walled Carbon Nanotubes Interactions with Mitochondrial Voltage-Dependent Anion Channels Using Molecular Docking and Perturbation Theory
Source: Sci Rep. 2017 Oct 16;7:13271. doi: 10.1038/s41598-017-13691-8 (PMC5643473; doi:10.1038/s41598-017-13691-8)
Supplement: Supplementary file 3 — Supplementary Figure SM03 [file 41598_2017_13691_MOESM3_ESM.docx]

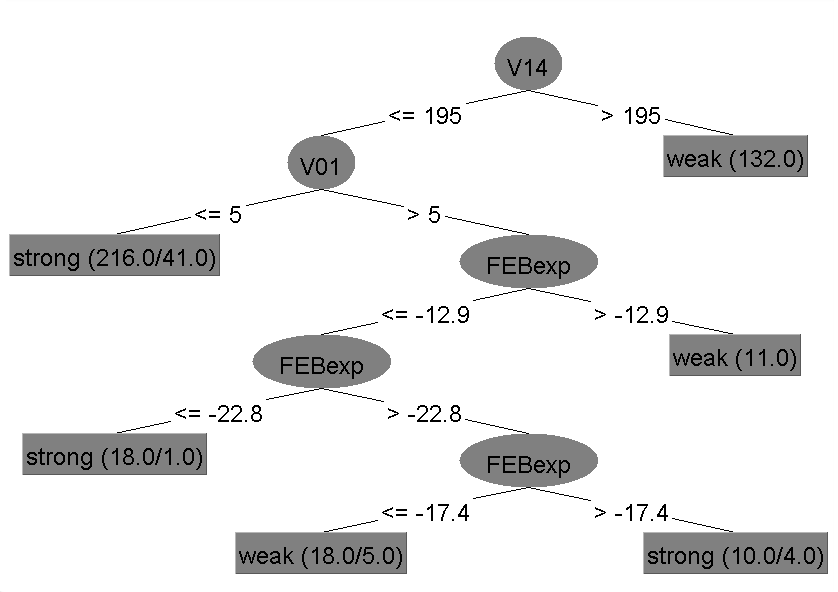


**SM03-A**. J48 tree classifiers using FS0 nanodescriptor dataset. The SWCNT-nanodescriptors represented in J48 decision tree are the frecuency of semi-empirical radial-breathing mode RBM (V14) from the Raman spectra, *n*-Hamada index (V1), and expected free energy of binding (FEB_exp_).


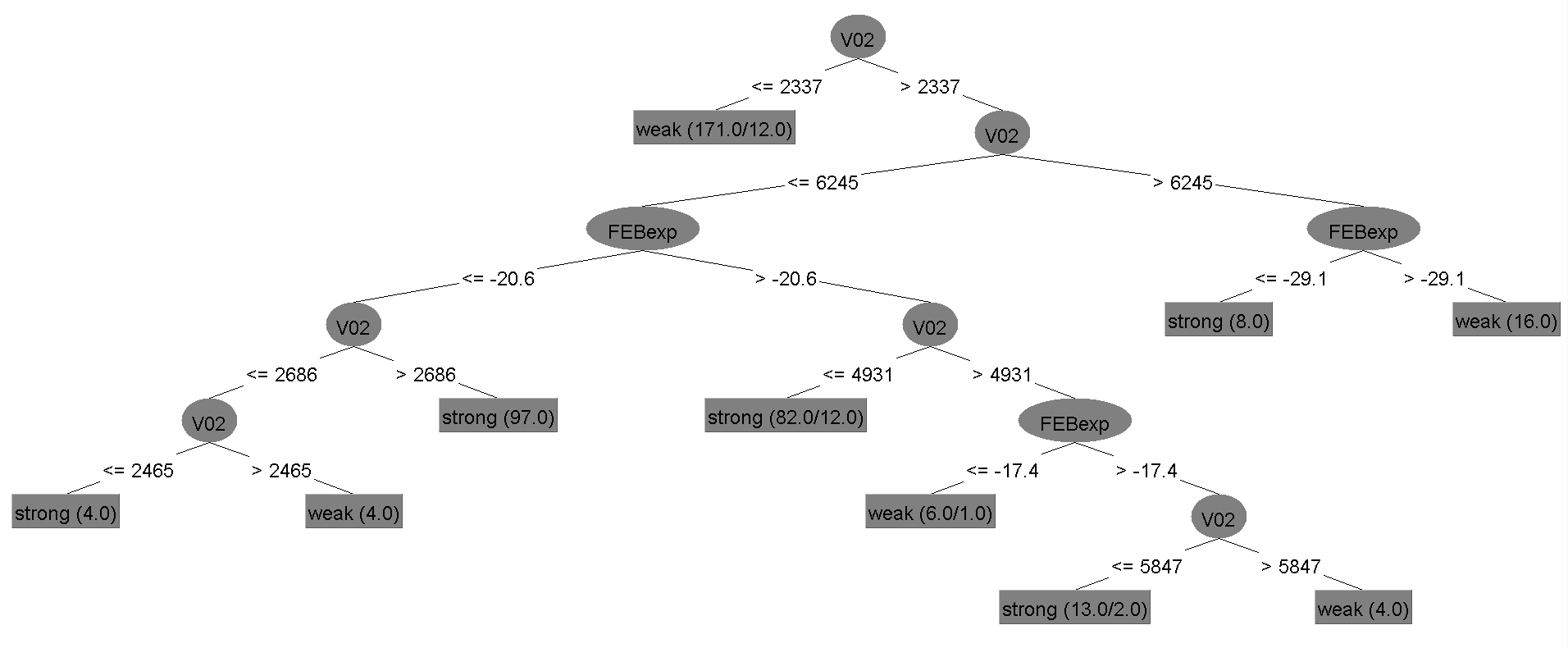


**SM03-B.** J48 tree classifiers using the minimum dataset FS3 based only on the expected free energy of binding (FEBexp) and SWCNT- Molecular mass (V02).
