# Supplementary material for: Decrypting Strong and Weak Single-Walled Carbon Nanotubes Interactions with Mitochondrial Voltage-Dependent Anion Channels Using Molecular Docking and Perturbation Theory
Source: Sci Rep. 2017 Oct 16;7:13271. doi: 10.1038/s41598-017-13691-8 (PMC5643473; doi:10.1038/s41598-017-13691-8)
Supplement: Supplementary file 4 — Supplementary Table SM04 [file 41598_2017_13691_MOESM4_ESM.docx]

**Table SM04**. Is presenting the performance of the classification nano-QSBR models using Weka tools. For each dataset, the results are ordered using 3 criteria: maximum AUROC, maximum TP Rate, minimum FP Rate. All the values are weighted averages for both classes (10-fold CV). Additional information is available in **SM04-1.xlsx**.

| **Weka Classifier** | **TPR** | **FPR** | **Prec** | **AUROC** | **Weka Classifier** | **TPR** | **FPR** | **Prec** | **AUROC** |
| --- | --- | --- | --- | --- | --- | --- | --- | --- | --- |
| **FS0 = FEB exp, V01,V11,V14** | | | | | **FS2 = FEB exp, V02, V03, V04, V09, V11, V12, V14** | | | | |
| K Star | 0.859 | 0.141 | 0.882 | 0.931 | Random Forest | 0.923 | 0.077 | 0.924 | 0.977 |
| Random Forest | 0.805 | 0.195 | 0.806 | 0.919 | K Star | 0.916 | 0.084 | 0.916 | 0.968 |
| Multilayer Perceptron | 0.854 | 0.146 | 0.871 | 0.896 | Multilayer Perceptron | 0.884 | 0.116 | 0.885 | 0.947 |
| REP Tree | 0.852 | 0.149 | 0.868 | 0.893 | REP Tree | 0.894 | 0.106 | 0.894 | 0.927 |
| JRip | 0.859 | 0.141 | 0.876 | 0.881 | J48 | 0.904 | 0.096 | 0.904 | 0.925 |
| Naïve Bayes | 0.830 | 0.171 | 0.870 | 0.881 | DecisionTable | 0.869 | 0.131 | 0.869 | 0.925 |
| J48 | 0.842 | 0.158 | 0.853 | 0.862 | Bayes Network | 0.830 | 0.171 | 0.870 | 0.925 |
| Bayes Network | 0.830 | 0.171 | 0.870 | 0.843 | JRip | 0.881 | 0.119 | 0.882 | 0.894 |
| Decision Table | 0.827 | 0.174 | 0.863 | 0.841 |  |  |  |  |  |
| **Pool = FEB exp, V00-V17** | | | | | **FS3 = new 8 Principal Components** | | | | |
| Random Forest | 0.916 | 0.084 | 0.916 | 0.973 | Random Forest | 0.872 | 0.128 | 0.872 | 0.956 |
| Multilayer Perceptron | 0.904 | 0.096 | 0.904 | 0.970 | Multilayer Perceptron | 0.899 | 0.101 | 0.899 | 0.955 |
| PART | 0.899 | 0.101 | 0.900 | 0.965 | JRip | 0.904 | 0.096 | 0.904 | 0.933 |
| K Star | 0.859 | 0.141 | 0.859 | 0.945 | REP Tree | 0.872 | 0.129 | 0.872 | 0.927 |
| REP Tree | 0.894 | 0.106 | 0.894 | 0.935 | K Star | 0.815 | 0.185 | 0.815 | 0.906 |
| DecisionTable | 0.864 | 0.136 | 0.864 | 0.929 | Bayes Net | 0.847 | 0.154 | 0.867 | 0.898 |
| J48 | 0.899 | 0.101 | 0.899 | 0.928 | J48 | 0.889 | 0.111 | 0.889 | 0.897 |
| JRip | 0.886 | 0.113 | 0.888 | 0.925 | Naïve Bayes | 0.840 | 0.161 | 0.864 | 0.888 |
| Bayes Network | 0.830 | 0.171 | 0.870 | 0.917 | DecisionTable | 0.842 | 0.158 | 0.848 | 0.880 |
| Naïve Bayes | 0.830 | 0.171 | 0.870 | 0.911 | OneR | 0.785 | 0.215 | 0.790 | 0.785 |
| OneR | 0.859 | 0.141 | 0.859 | 0.859 |  |  |  |  |  |
| **FS1 = FEB exp, V02, V03, V04, V10, V12** | | | | | **FS4 =FEB exp, V02** | | | | |
| Random Forest | 0.916 | 0.084 | 0.916 | 0.978 | Random Forest | 0.919 | 0.081 | 0.919 | 0.978 |
| K Star | 0.916 | 0.084 | 0.916 | 0.968 | PART | 0.914 | 0.086 | 0.915 | 0.962 |
| Multilayer Perceptron | 0.891 | 0.109 | 0.892 | 0.946 | K Star | 0.899 | 0.101 | 0.900 | 0.960 |
| J48 | 0.909 | 0.091 | 0.909 | 0.927 | IBk | 0.936 | 0.064 | 0.936 | 0.939 |
| REP Tree | 0.894 | 0.106 | 0.894 | 0.927 | J48 | 0.901 | 0.099 | 0.901 | 0.937 |
| IBk | 0.923 | 0.076 | 0.924 | 0.926 | Decision Table | 0.872 | 0.128 | 0.872 | 0.926 |
| Decision Table | 0.869 | 0.131 | 0.869 | 0.925 | Bayes Network | 0.859 | 0.141 | 0.867 | 0.919 |
| Bayes Network | 0.830 | 0.171 | 0.870 | 0.925 | REP Tree | 0.872 | 0.128 | 0.872 | 0.911 |
| Naïve Bayes | 0.827 | 0.174 | 0.861 | 0.886 | Multilayer Perceptron | 0.857 | 0.143 | 0.861 | 0.910 |
| JRip | 0.869 | 0.131 | 0.870 | 0.883 | JRip | 0.877 | 0.123 | 0.877 | 0.899 |
| OneR | 0.859 | 0.141 | 0.859 | 0.859 | OneR | 0.859 | 0.141 | 0.859 | 0.859 |
