# Supplementary material for: Decrypting Strong and Weak Single-Walled Carbon Nanotubes Interactions with Mitochondrial Voltage-Dependent Anion Channels Using Molecular Docking and Perturbation Theory
Source: Sci Rep. 2017 Oct 16;7:13271. doi: 10.1038/s41598-017-13691-8 (PMC5643473; doi:10.1038/s41598-017-13691-8)
Supplement: Supplementary file 5 — Supplementary Figure SM05 [file 41598_2017_13691_MOESM5_ESM.docx]

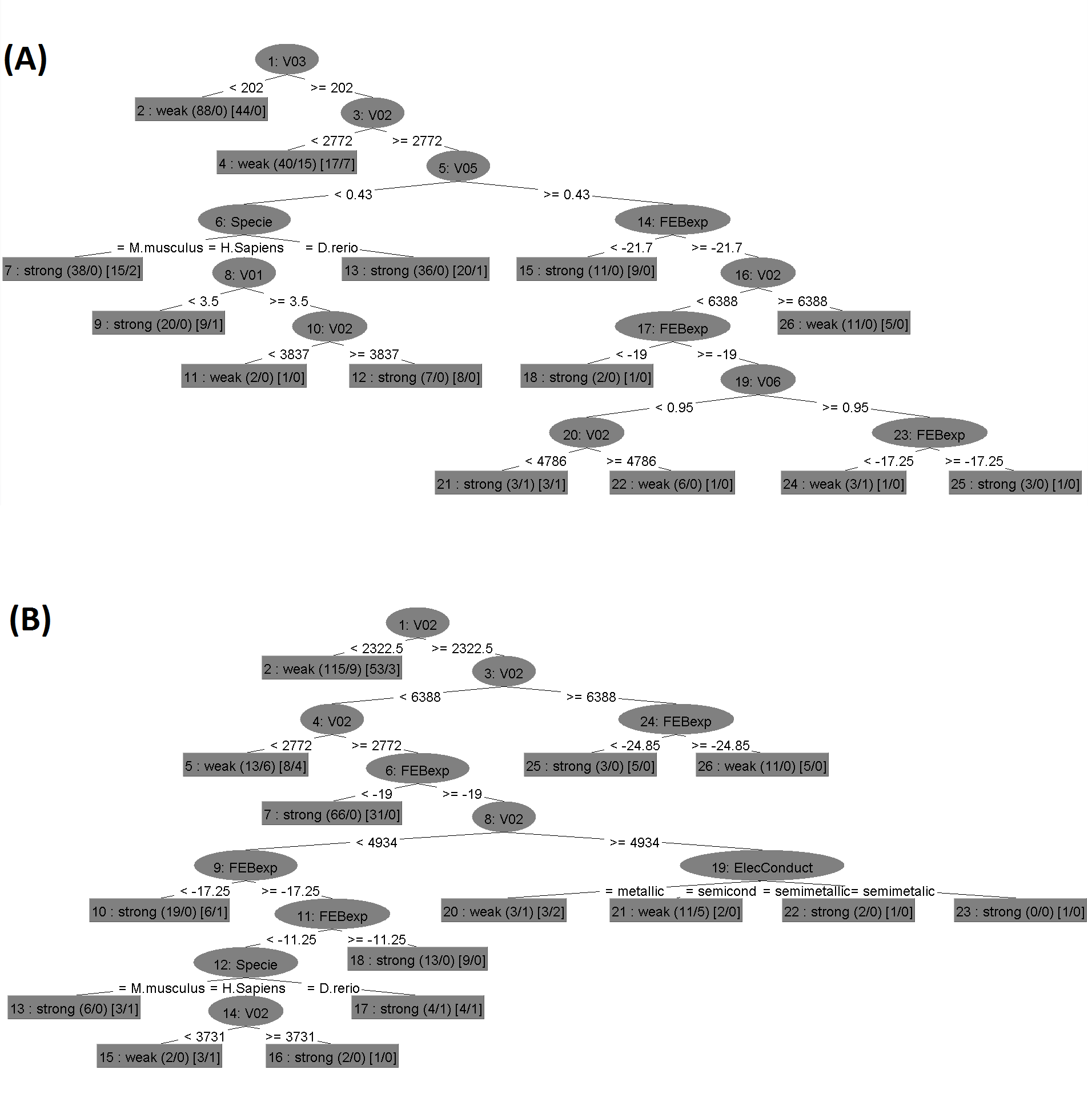


**SM05**. REP tree classifiers using extra information about the SWCNTs-parameters into the Pool dataset (**A**) and REP tree classifiers using extra information with minimum dataset FS4 (**B**).
