# Supplementary material for: Decrypting Strong and Weak Single-Walled Carbon Nanotubes Interactions with Mitochondrial Voltage-Dependent Anion Channels Using Molecular Docking and Perturbation Theory
Source: Sci Rep. 2017 Oct 16;7:13271. doi: 10.1038/s41598-017-13691-8 (PMC5643473; doi:10.1038/s41598-017-13691-8)
Supplement: Supplementary file 6 — Supplementary Table SM06 [file 41598_2017_13691_MOESM6_ESM.docx]

**SM06 Table**. Best Regression nano-QSBR models to predict FEB using RRegrs.

Additional information is available in **SM06.xlsx**.

| **Pool** | | | | | | |
| --- | --- | --- | --- | --- | --- | --- |
| **Method** | **SWCNT- Features *** | | **R^2^_train_*** | **RMSE_train_*** | **R^2^_test_*** | **RMSE_test_*** |
| RF | 19 | | 0.801 | 0.0914 | 0.833 | 0.0844 |
| RF-RFE | 3 | | 0.775 | 0.1009 | 0.798 | 0.0953 |
| NN | 19 | | 0.750 | 0.1057 | 0.757 | 0.1059 |
| SVM-RFE | 12 | | 0.556 | 0.1426 | 0.486 | 0.1532 |
| LM | 19 | | 0.472 | 0.1538 | 0.409 | 0.1617 |
| GLM | 19 | | 0.465 | 0.1549 | 0.404 | 0.1626 |
| Lasso | 16 | | 0.478 | 0.1526 | 0.403 | 0.1624 |
| PLS | 19 | | 0.435 | 0.1588 | 0.400 | 0.1625 |
| ENET | 16 | | 0.469 | 0.1540 | 0.398 | 0.1631 |
| **LDA classification split**** | | | | | | |
| **Method** | **SWCNT-Features** | **R^2^_train_** | | **RMSE_train_** | **R^2^_test_** | **RMSE_test_** |
| RF | 19 | 0.816 | | 0.0889 | 0.822 | 0.0877 |
| NN | 19 | 0.773 | | 0.1009 | 0.728 | 0.1121 |
| GLM | 19 | 0.429 | | 0.1606 | 0.491 | 0.1483 |
| **LDA classification features & split**** | | | | | | |
| **Method** | **SWCNT-Features** | **R^2^_train_** | | **RMSE_train_** | **R^2^_test_** | **RMSE_test_** |
| RF**** | 4 | 0.673 | | 0.1204 | 0.672 | 0.1193 |
| NN | 4 | 0.654 | | 0.1236 | 0.661 | 0.1215 |
| GLM | 4 | 0.419 | | 0.1606 | 0.433 | 0.1564 |

Note: All datasets are normalized; No RRegrs filter have been used (all SWCNT-nanodescriptors are used); * averaged values using standard 10 splits of RRegrs methodology; ** only one split used to obtain the LDA classification model, all SWCNT-nanodescriptors; *** only one split used to obtain the LDA classification model and only the SWCNT-nanodescriptors from the LDA classification nanoQSBR-model (FEBexp, V01, V11, and V14); **** 300 trees.
