# Supplementary material for: Decrypting Strong and Weak Single-Walled Carbon Nanotubes Interactions with Mitochondrial Voltage-Dependent Anion Channels Using Molecular Docking and Perturbation Theory
Source: Sci Rep. 2017 Oct 16;7:13271. doi: 10.1038/s41598-017-13691-8 (PMC5643473; doi:10.1038/s41598-017-13691-8)
Supplement: Supplementary file 7 — Supplementary Figure SM07 [file 41598_2017_13691_MOESM7_ESM.docx]

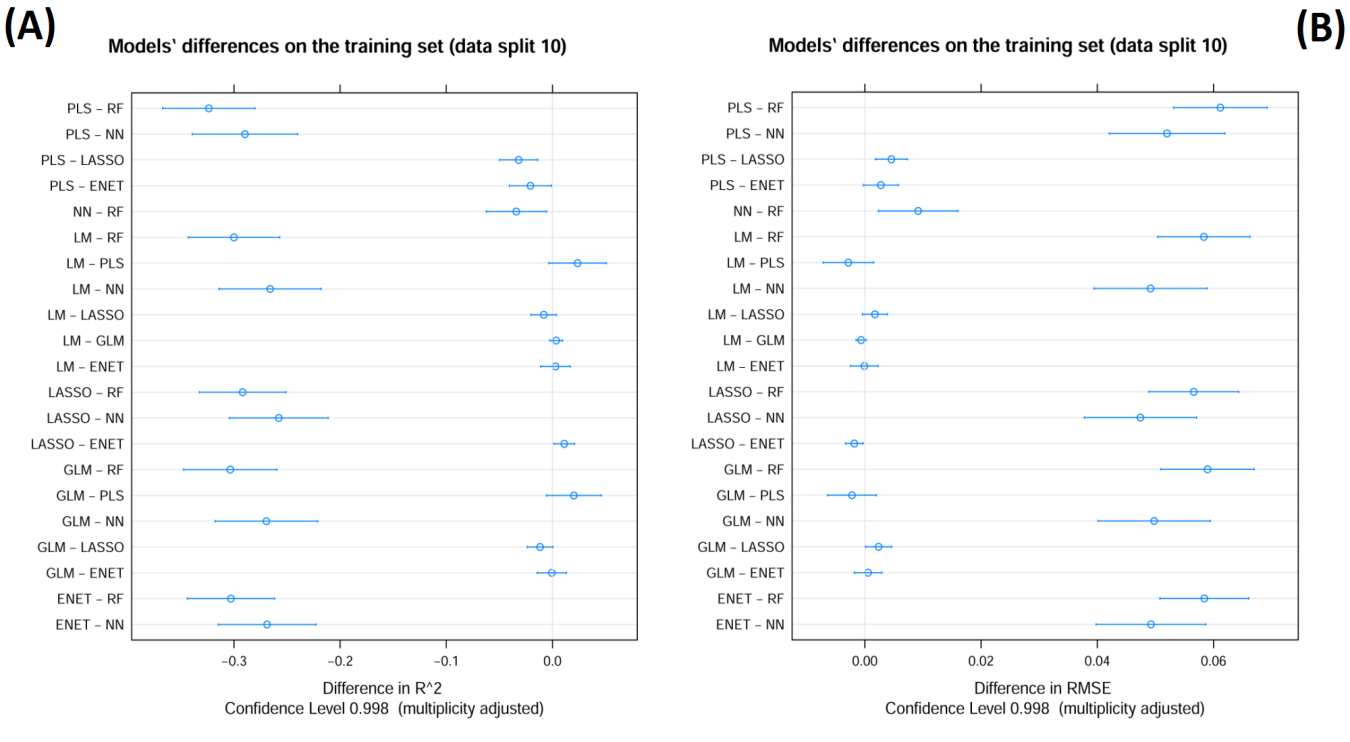


**SM07**. **RRegrs pairwise model comparisons of (A) R^2^_test_ and (B) RMSE_test_ for prediction of SWCNTs – VDACs free energy of binding (FEB as nanotoxicity)**. The average performance value (dot) with two-sided confidence limits as computed by Student’s t-test with Bonferroni multiplicity correction
