# Supplementary material for: Decrypting Strong and Weak Single-Walled Carbon Nanotubes Interactions with Mitochondrial Voltage-Dependent Anion Channels Using Molecular Docking and Perturbation Theory
Source: Sci Rep. 2017 Oct 16;7:13271. doi: 10.1038/s41598-017-13691-8 (PMC5643473; doi:10.1038/s41598-017-13691-8)
Supplement: Supplementary file 8 — Supplementary Figure SM08 [file 41598_2017_13691_MOESM8_ESM.docx]

**
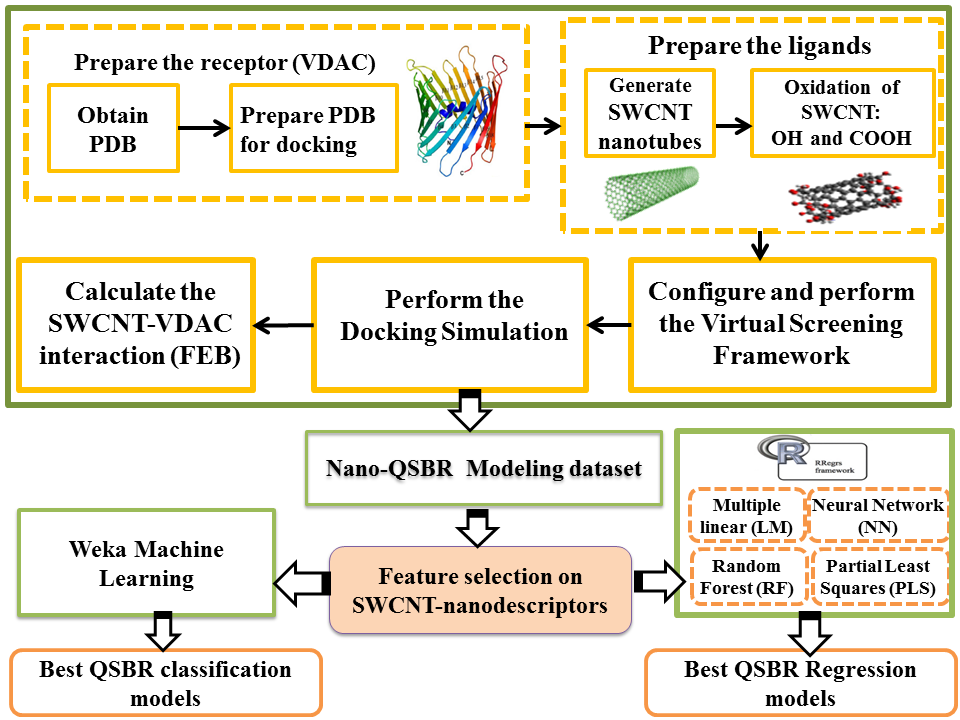
**

**SM08**. **General flowchart of the NQSBR performed from the molecular docking simulations and virtual screening to study the SWCNT–VDAC binding affinity.** PDB: protein data bank (<http://www.rcsb.org/pdb/home/home.do>)^49^. SWCNT: single-walled carbon nanotube. QSBR: Quantitative Structure Binding Relationship.
